# Safety and efficacy of a single intra-articular injection of a novel enhanced protein solution (JTA-004) compared to hylan G-F 20 in symptomatic knee osteoarthritis: a randomized, double-blind, controlled phase II/III study

**DOI:** 10.1186/s12891-021-04750-3

**Published:** 2021-10-19

**Authors:** Marie Bettonville, Marc Léon, Joëlle Margaux, Didier Urbin-Choffray, Emilie Theunissen, Tatiana Besse-Hammer, Yves Fortems, Séverine Verlinden, Olivier Godeaux, Anne-Sophie Delmarcelle, Jean-François Kaux

**Affiliations:** 1 Bone Therapeutics S.A, Rue Auguste Piccard 37, 6041 Gosselies, Belgium; 2 Current Address: iTeos Belgium SA, Rue des Frères Wright 29, 6041 Gosselies, Belgium; 3 CHU Ambroise Paré, Boulevard Kennedy 2, 7000 Mons, Belgium; 4 CUB Erasme, Route de Lennik 808, 1070 Bruxelles, Belgium; 5 CHR Citadelle, Boulevard du 12ème de Ligne 1, 4000 Liège, Belgium; 6 Clinique Saint-Pierre Ottignies, Avenue Reine Fabiola 9, 1340 Ottignies, Belgium; 7 CHU Brugmann, Place Arthur Van Gehuchten 4, 1020 Bruxelles, Belgium; 8 AZ Sint-Jozef, Oude Liersebaan 4, 2390 Malle, Belgium; 9 CHR Haute Senne, Chaussée de Braine 49, 7060 Soignies, Belgium; 10 Current Address: ZAM Consulting, Avenue de la Calèche 24, 1300 Wavre, Belgium; 11 Current Address: Cryotherapeutics SA, Rue de Bruxelles 174 E40 Business Park, 4340 Awans, Belgium; 12 Physical and Rehabilitation Medicine & Sport Traumatology Department, University and University Hospital of Liège, Avenue de l’Hôpital 35, 4000 Liège, Belgium

**Keywords:** Knee osteoarthritis, Clinical trial, Intra-articular injection, Hyaluronic acid, Clonidine, Human plasma

## Abstract

**Background:**

New minimally invasive treatments are vital to delay joint replacement surgery in patients with knee osteoarthritis. This study was designed to select the most effective among three formulations of an enhanced protein solution containing clonidine, hyaluronic acid, and human plasma (JTA-004), and compare the safety and efficacy of intra-articular administration of the selected formulation with a reference treatment (hyaluronic acid) in symptomatic knee osteoarthritis patients.

**Methods:**

In this two-stage, double-blind, phase II/III study conducted in 12 Belgian centers, 50–79-year-old patients with primary knee osteoarthritis were randomized (1:1:1:1) to receive one dose of one of three JTA-004 formulations (differing in clonidine concentration [50 or 100 μg/ml] and volume [2 or 4 ml]) or the reference treatment (hylan G-F 20). Patients were evaluated using Western Ontario McMaster Universities (WOMAC®) Scores and the Short-Form health survey up to 6 months post-injection (Month 6). Drug consumption and safety were evaluated.

**Results:**

Among 164 treated patients, 147 completed the study. The JTA-004 formulation containing 200 μg clonidine and 20 mg hyaluronic acid in 2 ml (JTA-200/2) was selected based on interim results at Month 6. The difference in adjusted mean change in WOMAC Pain Subscale Score from baseline (JTA-200/2 minus reference group) at Month 6 was − 9.49 mm; statistical superiority of JTA-200/2 over the reference was not demonstrated. No statistically significant differences in adjusted mean changes from baseline between JTA-200/2 and reference groups were observed for Pain, Physical Function and Stiffness Subscales WOMAC Scores, Total WOMAC Score, and Well-being Score at any timepoint, although JTA-200/2 induced larger improvements in WOMAC Scores than the reference. Statistically significantly larger improvements in WOMAC Pain Subscale Scores for JTA-004 versus the reference were observed in *post-hoc* analyses on pooled data from all JTA-004 formulations at Month 6 (*p* = 0.030) and Month 3 (*p* = 0.014). All JTA-004 formulations had clinically acceptable safety profiles.

**Conclusions:**

This study provided preliminary evidence of the safety of intra-articular injection of JTA-004 in knee osteoarthritis patients. Phase III randomized controlled trials with larger sample sizes are needed to evaluate the efficacy of JTA-004 in knee osteoarthritis.

**Trial registration:**

Clinicaltrials.gov/identifier NCT02740231;  clinicaltrialsregister.eu/identifier 2015–002117-30. Retrospectively registered 13/4/2016.

**Supplementary Information:**

The online version contains supplementary material available at 10.1186/s12891-021-04750-3.

## Background

Osteoarthritis (OA) is a degenerative, chronic, and progressive joint disease with a multifactorial etiology and is most common in weight-bearing joints, such as knees [1]. Currently, no treatment is available to stop OA progression, and joint replacement surgery is the only solution for severe cases. Non-operative treatment options include intra-articular drug injections into affected joints, which increase local bioavailability and reduce systemic exposure, adverse events (AEs), and costs compared with traditional pharmacologic therapies [2–4]. Intra-articular injections of corticosteroids having anti-inflammatory properties [5], and of hyaluronic acid (HA), a viscosupplement with analgesic, anti-inflammatory, and potential disease-modifying properties [6, 7], have been widely used [2, 3]. However, conclusions regarding their clinical utility are inconsistent, repeated injections are needed, long-term effects remain unclear, and they may be associated with AEs [3, 4]. Recently, biological treatments targeting key biochemical pathways have been developed, such as autologous platelet-rich plasma (PRP) injections to enhance tissue regeneration [8]. PRP injections were shown to potentially improve pain and function compared to HA or placebo injections in patients with knee OA, but their frequent use increased the risk of AEs, and PRP preparations vary considerably [9–13]. Therefore, other minimally invasive therapeutic options are needed for the treatment of knee OA [14].

In this context, JTA-004 (Bone Therapeutics S.A., Belgium), an enhanced protein solution derived from human plasma that contains clonidine and HA obtained via bacterial fermentation, has been developed. The local administration of JTA-004 into the joint cavity of patients with osteoarthritis is intended (i) to relieve local pain and discomfort associated with intra-articular injections through the short-term analgesic properties of clonidine [15, 16], and (ii) to restore the joint homeostasis thanks to the interaction between human plasma and HA. Once injected in the knee joint, human plasma induces jellification through the coagulation cascade and forms a clotting gel resulting in a tridimensional network stabilized by interactions between HA fibers and the patient’s synovial proteins [17]. This gel presents a mechanical and rheological behavior close to the synovial fluid with both lubrication and shock damping effect, offering protection of the patient’s cartilage (unpublished results).

The first objective of this study was to select the most effective among three JTA-004 formulations containing the same components at different dosages and volumes. The second objective was to compare the safety and efficacy of a single intra-articular administration of the selected JTA-004 formulation with a reference HA treatment (hylan G-F 20, Synvisc-One®, Sanofi, France) during 6 months in symptomatic knee OA patients. Hylan G-F 20 was chosen as reference since it was shown to be safe and effective, and it provided statistically significant and clinically relevant pain relief in patients with knee OA [18–20].

## Methods

### Study design

This two-stage, prospective, multicenter, randomized, double-blind, controlled phase II/III study was conducted in 12 Belgian centers from 24 March 2016 (first visit of first patient) to 27 April 2018 (last visit of last patient). It was a two-stage study, with an interim analysis to be performed when 116 patients, i.e., 104 patients with available data considering a 10% drop-out, had been followed up for 3 months or were discontinued from the study. Unblinded safety and efficacy data were assessed by an Independent Data Monitoring Committee (IDMC). The interim analysis was planned to re-assess the sample size and stop the trial for futility or important safety concerns if necessary.

The study was performed in accordance with the current version of the Declaration of Helsinki (Fortaleza, Brazil, October 2013) and the International Conference on Harmonization Good Clinical Practice Guideline. The study protocol, its amendments, and the patient information sheet were reviewed and approved by the appropriate independent Ethics Committees. This study was registered at clinicaltrials.gov (NCT02740231) and clinicaltrialsregister.eu (EudraCT number: 2015–002117-30).

### Study population

Eligible participants were 50–79-year-old men and women diagnosed with primary knee OA, who were able to walk unassisted (crutch/walking stick use was allowed), had previous insufficient/failed response to analgesics and/or non-steroidal anti-inflammatory drugs (NSAIDs), were willing and able to abstain from knee physical therapy and braces during the study, and had a body mass index (BMI) < 35.

Eligible patients had to fulfill the following American College of Rheumatology criteria: pain ≥40 mm on a 0–100 mm Visual Analogue Scale (VAS) during 3 days preceding the screening visit, morning stiffness ≤30 min, and Kellgren-Lawrence grade II or III [21, 22]. The list of exclusion criteria is given in Additional file 1, Supplementary Text 1.

### Interventions

After signing the Informed Consent Form, eligible patients were randomized (1:1:1:1) to receive one of the three evaluated JTA-004 formulations (JTA-100/2, JTA-200/2 and JTA-200/4 groups) or the reference treatment (hylan G-F 20 in 6 ml, reference group). The three JTA-004 formulations differed in clonidine concentration (50 or 100 μg/ml) and volume of injection (2 or 4 ml) (Table 1). They were provided as a freeze-dried powder for solution that needs to be reconstituted with sterile water before single intra-articular injection. The reference treatment (hylan G-F 20) was a sterile viscoelastic solution provided in a ready-to-use syringe. The solution (6 ml) contained 48 mg sodium hyaluronate.

**Table 1:** Description of the three administered JTA-004 formulations

JTA-004 formulation	Plasma protein solution		Clonidine		HA		Volume of injection
	Concentration	Amount	Concentration	Amount	Concentration	Amount	
**JTA-100/2**	1.02 g/ml	2.04 g	50 μg/ml	100 μg	10 mg/ml	20 mg	2 ml
**JTA-200/2**	1.02 g/ml	2.04 g	100 μg/ml	200 μg	10 mg/ml	20 mg	2 ml
**JTA-200/4**	1.02 g/ml	4.08 g	50 μg/ml	200 μg	10 mg/ml	40 mg	4 ml

*HA* hyaluronic acid

Although the use of the lateral midpatellar portal approach results in high accuracy rates of needle placement into the intra-articular space [23], the investigators could use another knee portal. The injection was accomplished following puncture through the skin and into the joint space. The use of local anesthesia prior to the intra-articular injection was at the discretion of the independent physician. If effusion was found in the knee upon needle placement in the joint space, it had to be removed before the injection. After withdrawing the needle from the joint space, light pressure was applied to the injection site, followed by application of a simple adhesive bandage. The patient remained for 45 min post-injection under clinical and blood pressure monitoring. Patients were advised to wait until the next day before returning to normal activities. For post-administration pain management, it was recommended that patients rest and apply ice to the injection site.

### Outcomes

Efficacy and safety were evaluated on the injection day and approximately 2 weeks, 3 months, and 6 months after. At each follow-up visit, well-validated Western Ontario McMaster Universities (WOMAC®) VA3.1 Osteoarthritis Scores were determined using self-administered, patient-centered health status questionnaires allowing a thorough evaluation of pain, stiffness, and knee function (24 questions through three subscales) [24]. VAS were used in the WOMAC questionnaires, on which patients had to mark points on horizontal lines to represent their symptoms’ perceptions (from no symptoms [0 mm] to extreme symptoms [100 mm]). The WOMAC Scores were determined by measuring the distances from the left-hand end of the lines to the points that the patients marked. The minimal clinically important difference (MCID) was defined as 20% or 10 mm of the baseline [24, 25]. The primary objective in the first study stage was to select the most effective JTA-004 formulation by comparing differences between each JTA group and the reference group in adjusted mean changes from baseline at Month 3 in WOMAC Pain Subscale Score. The primary objective in the second study stage was to demonstrate superiority of the efficacy of the selected formulation over the reference. JTA-004 would be proven to be superior if the upper bound of the 95% confidence interval (CI) of the difference in adjusted mean change in WOMAC Pain Subscale Score from baseline at Month 6 between the selected JTA and the reference groups was < 0.

Secondary endpoints included the WOMAC Total Score over time and the WOMAC Pain Subscale Score at Month 3 (Additional file 2, Supplementary Table 1). Exploratory endpoints included the WOMAC Total Score at Month 6, the WOMAC Pain Subscale Score over time, the WOMAC Physical Function Subscale and the WOMAC Stiffness Subscale Scores, Well-Being scores estimated by the Short Form Health Survey (SF-12 questionnaire, the abridged form of the SF-36 questionnaire taking into account physical elements, psychological aspects, and a subjective health perception [26]), and consumption of analgesics and NSAIDs reported on patients’ open questionnaires at Month 6 and over time.

The safety endpoints included the evaluation of the occurrence of AEs and serious adverse events (SAEs) reported on patients’ open questionnaires, related or not to the product or procedures, abnormal laboratory results in terms of hematology, serum chemistry, and coagulation parameters, and clinically relevant findings at physical examination (including vital signs) during the entire study duration. Treatment-related AEs included AEs for which the investigator answered “Yes” or “Possibly” to relationship with study treatment/procedures on the electronic Case Report Form and AEs with missing/unknown relationship.

### Randomization

An Interactive Web Response System was used to perform the randomization.

### Blinding

Investigators (who recruited, included, and assessed patients) and patients were blind to treatment assignments. Only local pharmacists and the independent physicians performing the intra-articular injections were unblind.

### Sample size

Based on published data [25, 27–29], the scenario of a mean between-group difference of − 7 mm for the change in WOMAC Pain Subscale from baseline at Month 6 (i.e., JTA-004 was better than the reference treatment by a mean of 7 mm) with a standard deviation (SD) of 10.5 mm was assumed, which required 37 patients per group to reach a power of 80% to test for superiority, keeping the type I error at 0.05 (two-sided). With an estimated drop-out level of 10%, 41 patients per group were planned to be included.

Based on the differences and associated variability in mean change from baseline in WOMAC Pain Subscale Score between the selected JTA-004 formulation and the reference treatment observed at interim analysis, the IDMC determined that the target sample size should be re-estimated in order to avoid underpowering the study results at the final analysis. They recommended to increase the sample size to 76 participants per group. However, it was decided that no additional participants would be recruited.

### Statistical analyses

Selection of the most effective JTA-004 formulation at the interim analysis was based on the differences between each JTA group and the reference group in mean changes from baseline in WOMAC Pain Subscale Score at Month 3. Differences were determined by analyses of covariance (ANCOVA) adjusted for differences in baseline values. Their CIs were calculated using the Dunnett’s test procedure to control the experiment-wise error probability at 0.05 [30]. Because the analysis of data at Month 3 was inconclusive, available data at Month 6 were also reviewed at interim analysis, as pre-specified in the statistical analysis plan.

At final analysis, the following pre-defined analyses were performed: mean changes in WOMAC Pain Subscale, WOMAC Total, WOMAC Physical Function and Stiffness Subscales, and SF-12 Well-Being Scores between baseline and Month 6 and Month 3 were compared between the selected JTA-004 formulation and the reference group by ANCOVA analyses adjusted for baseline values (treatment group as fixed factor and baseline value as covariate). To evaluate changes over time, a Mixed-effect Model for Repeated Measurements was also used. To account for multiple comparisons between interim and final analyses and control the experiment-wise error probability at 0.05, adjusted 95% CIs and *p*-values at final analysis were calculated using the Dunnett-corrected t-value [30]. Tests of the secondary endpoints were performed in a sequential hierarchical manner based on Hochberg’s step-up-method at the two-sided significance level of 0.050 and 0.025 for each of the two secondary endpoints, respectively. If superiority could not be demonstrated under the statistical testing strategy for secondary endpoints, nominal p-values were provided for information only. For exploratory endpoints, p-values were also provided for information only.

To further explore potentially clinically meaningful treatment effects, *post-hoc* exploratory analyses were performed to compare the non-selected JTA groups and the pooled data from all three JTA groups to the reference group.

Missing values due to partially non-completed WOMAC questionnaires were imputed as per the WOMAC Osteoarthritis Index User Guide VII. Missing values for efficacy variables were not accounted for in the primary analysis; however, supportive analyses in which missing data were imputed as per a pre-defined algorithm were performed.

Statistical analyses were performed using SAS® software version 9.2 (SAS Institute, Cary, NC, USA).

## Results

### Study population

**Fig. 1:**
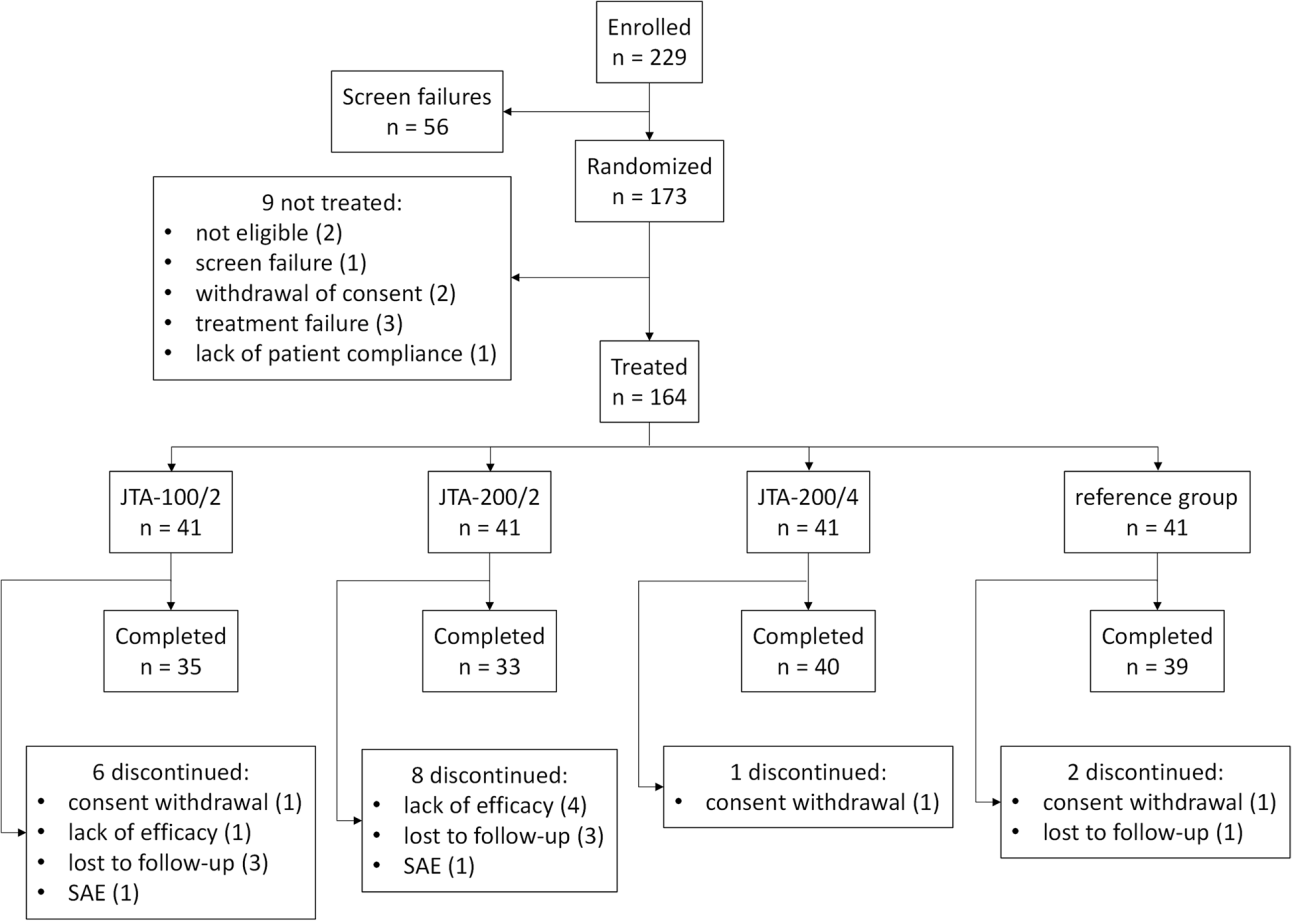
Patient disposition. JTA-100/2, group of patients receiving an injection of JTA-004 with 100 μg clonidine and 20 mg hyaluronic acid; JTA-200/2, group of patients receiving an injection of JTA-004 with 200 μg clonidine and 20 mg hyaluronic acid; JTA-200/4, group of patients receiving an injection of JTA-004 with 200 μg clonidine and 40 mg hyaluronic acid; reference group, group of patients receiving an injection of the reference treatment (hylan G-F 20); n, number of patients; SAE, serious adverse event

Of the 229 screened patients, 173 had to be randomized to reach a total number of 164 treated patients (41 per group as planned per-protocol). Of these, 147 patients completed the study and attended the visit at Month 6 (Fig. 1). Since all patients were treated as assigned by randomization scheme, the Full Analysis Set (FAS, all randomized and treated patients) was identical to the Safety Set (all treated patients).

The mean age at enrollment was 62.7 years, and 68.3% of patients were women (Table 2). The mean height, weight, and BMI were 1.66 m, 79.2 kg, and 28.5 kg/m^2^. Overall, 55.5 and 44.5% of patients had knee OA with a Kellgren-Lawrence grade II and III. The baseline demographic characteristics of the study participants were mostly comparable between groups.

**Table 2:** Demographic characteristics (Full Analysis Set)

		JTA-100/2 (***N*** = 41)	JTA-200/2 (N = 41)	JTA-200/4 (N = 41)	reference (N = 41)	overall (***N*** = 164)
Age (years)	Mean ± SD	64.2 ± 8.0	61.7 ± 7.0	62.9 ± 7.2	61.8 ± 7.7	62.7 ± 7.5
Sex	Male, n (%)	10 (24.4)	17 (41.5)	14 (34.1)	11 (26.8)	52 (31.7)
	Female, n (%)	31 (75.6)	24 (58.5)	27 (65.9)	30 (73.2)	112 (68.3)
Height (m)	Mean ± SD	1.65 ± 0.08	1.68 ± 0.11	1.66 ± 0.10	1.66 ± 0.10	1.66 ± 0.10
Weight (kg)	Mean ± SD	79.4 ± 13.8	83.7 ± 13.9	77.5 ± 13.8	76.2 ± 15.9	79.2 ± 14.5
BMI (kg/m^2^)	Mean ± SD	29.0 ± 3.9	29.6 ± 3.5	28.0 ± 3.6	27.5 ± 4.2	28.5 ± 3.9
Kellgren-Lawrence grade	II, n (%)	18 (43.9)	23 (56.1)	25 (61.0)	25 (61.0)	91 (55.5)
	III, n (%)	23 (56.1)	18 (43.9)	16 (39.0)	16 (39.0)	73 (44.5)

*BMI* body mass index, *JTA-100/2* group of patients receiving an injection of JTA-004 with 100 μg clonidine and 20 mg hyaluronic acid, *JTA-200/2* group of patients receiving an injection of JTA-004 with 200 μg clonidine and 20 mg hyaluronic acid, *JTA-200/4* group of patients receiving an injection of JTA-004 with 200 μg clonidine and 40 mg hyaluronic acid; n (%), number (percentage) of patients; N, total number of patients; reference, group of patients receiving an injection of the reference treatment (hylan G-F 20); SD, standard deviation

Treatment administration was well-balanced between left and right knees, and mostly performed through lateral midpatellar and anterolateral approaches. Synovial fluid was aspirated before injection in 29 patients (mean volume [SD]: 4.1 ml [6.7]). In one patient (reference group), the physician could not inject the entire treatment volume (5 ml instead of 6 ml).

### Efficacy results

#### Selection of the most effective JTA-004 formulation (interim analysis)

In the interim analysis, 116 patients were included (30, 29, 29, and 28 in the JTA-100/2, JTA-200/2, JTA-200/4, and reference groups). The difference between each JTA group and the reference group in mean change from baseline in WOMAC Pain Subscale Score adjusted for baseline values was in favor of the JTA groups at Month 3 (Table 3). The difference was largest for the JTA-200/4 group. The difference was still in favor of JTA groups for all formulations at Month 6, with the largest difference observed for the JTA-200/2 group. The same pattern, i.e., largest difference for the JTA-200/4 group at Month 3 and the JTA-200/2 group at Month 6, was observed for the WOMAC Total and Physical Function Subscale Scores (Additional file 2, Supplementary Tables 2 and 3). Since the primary endpoint in the second study stage was the difference in WOMAC Pain Subscale Score at Month 6, the IDMC members based their decision on trends observed at Month 6 and selected the JTA-200/2 formulation as the most effective with respect to the predefined decision rules. The IDMC concluded that there was no marked difference between treatment groups regarding the occurrence of AEs indicating safety concerns, and they recommended to continue the study.

**Table 3:** Results of the interim analysis to select the most effective JTA formulation (Full Analysis Set)

Difference between each JTA and the reference group in adjusted mean change from baseline in WOMAC Pain Subscale Score	JTA-100/2	JTA-200/2	JTA-200/4
**Month 3**			
N	28	24	29
Adjusted Mean (SE)	−11.79 (6.32)	−9.50 (6.60)	−16.50 (6.28)
Adjusted CI (a)	−26.87, 3.29	−25.25, 6.24	−31.48, − 1.53
p-value	0.160	0.344	0.027
**Month 6**			
N	22	19	24
Adjusted Mean (SE)	−8.10 (7.03)	−11.22 (7.29)	−7.37 (6.94)
Adjusted CI (a)	−24.94, 8.74	− 28.68, 6.24	−24.01, 9.27
p-value	0.526	0.295	0.588

*CI* confidence interval, *JTA-100/2* group of patients receiving an injection of JTA-004 with 100 μg clonidine and 20 mg hyaluronic acid, *JTA-200/2* group of patients receiving an injection of JTA-004 with 200 μg clonidine and 20 mg hyaluronic acid, *JTA-200/4* group of patients receiving an injection of JTA-004 with 200 μg clonidine and 40 mg hyaluronic acid, *N* total number of patients, *SE* standard error, *WOMAC* Western Ontario McMaster Universities

(a) calculated using the Dunnett’s test procedure (overall type-I error rate of 0.05)

Differences in adjusted mean change from baseline in WOMAC Pain Subscale Score were evaluated using an ANCOVA model with treatment group as fixed factor and baseline value of WOMAC Pain Subscale Score as covariate

#### WOMAC pain subscale score (final analysis)

**Fig. 2:**
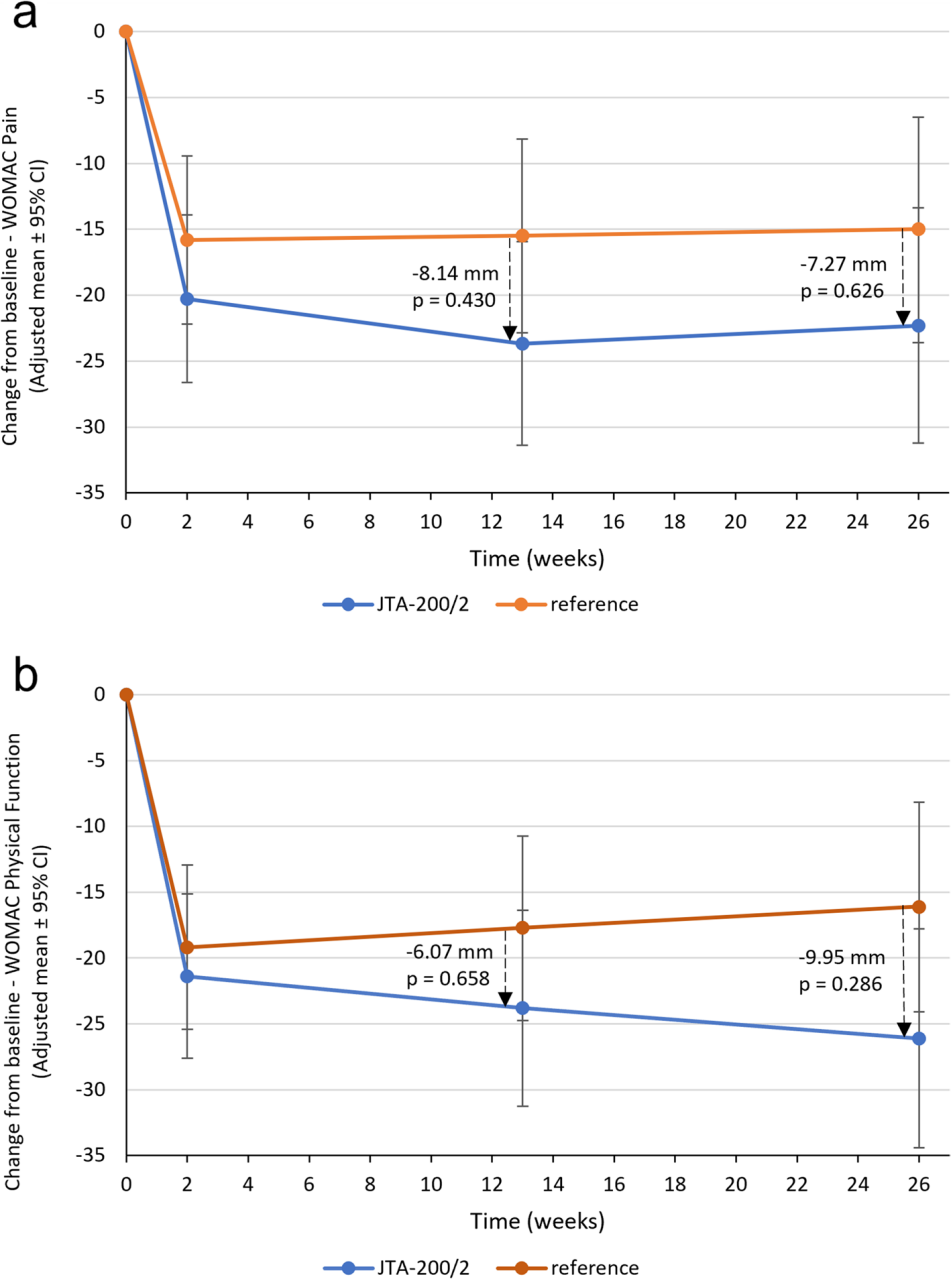
Change from baseline in WOMAC Subscale Scores over time (Full Analysis Set). CI, confidence interval (calculated using Dunnett-corrected t-value); JTA-200/2, group of patients receiving an injection of JTA-004 with 200 μg clonidine and 20 mg hyaluronic acid; reference, group of patients receiving an injection of the reference treatment (hylan G-F 20); WOMAC, Western Ontario McMaster Universities. Panel **a**: Change from baseline in WOMAC Pain Subscale Score. Panel **b**: Change from baseline in WOMAC Physical Function Subscale Score. Changes from baseline in WOMAC Subscale Scores over time were evaluated using a Mixed-effect Model for Repeated Measurements with absolute change from baseline to the visit in WOMAC Subscale Score as response variable, treatment group and visit as factors, baseline WOMAC Subscale Score as covariate and treatment group-visit interaction

In the FAS, mean WOMAC Pain Subscale Scores at baseline were 56.2 mm (SD: 20.6) and 46.5 mm (19.7) in the JTA-200/2 (selected JTA formulation) and reference groups. Observed mean WOMAC Pain Subscale Scores at subsequent visits were lower than at baseline in both groups (over time analysis; Fig. 2a).

**Fig. 3:**
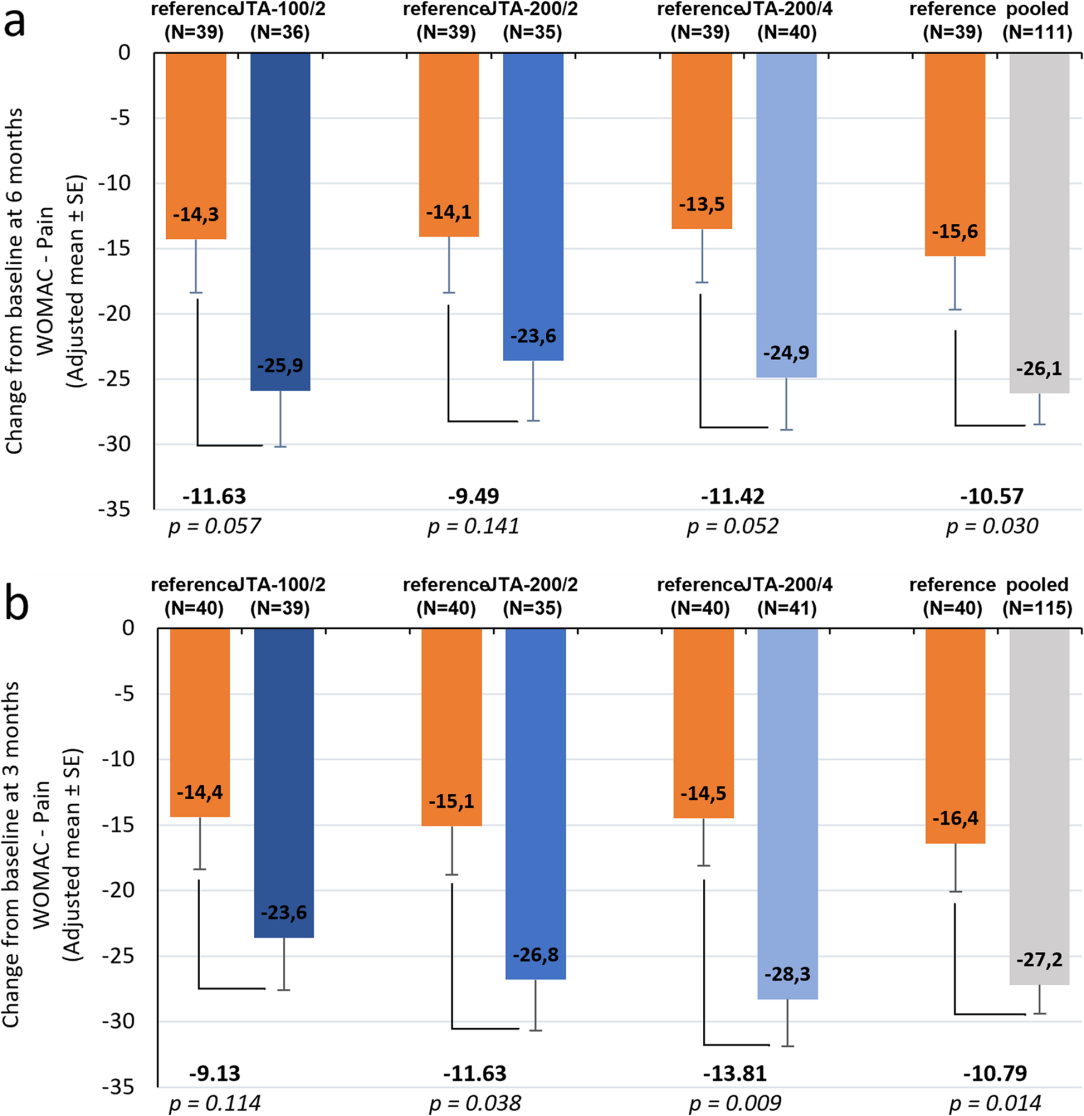
Difference in adjusted mean change from baseline in WOMAC Pain Subscale Score (Full Analysis Set). JTA-100/2, group of patients receiving an injection of JTA-004 with 100 μg clonidine and 20 mg hyaluronic acid; JTA-200/2, group of patients receiving an injection of JTA-004 with 200 μg clonidine and 20 mg hyaluronic acid; JTA-200/4, group of patients receiving an injection of JTA-004 with 200 μg clonidine and 40 mg hyaluronic acid; pooled, group of patients receiving an injection of any formulation of JTA-004; reference, group of patients receiving an injection of the reference treatment (hylan G-F 20); N, number of patients; SE, standard error; WOMAC, Western Ontario McMaster Universities. Panel a: difference at Month 6. Panel b: difference at Month 3. Differences in adjusted mean change from baseline in WOMAC Pain Subscale Score were evaluated using an ANCOVA model with treatment group as fixed factor and baseline value of WOMAC Pain Subscale Score as covariate

At Month 6, adjusted mean changes in WOMAC Pain Subscale Score from baseline were − 23.6 mm (standard error [SE]: 4.6) and − 14.1 mm (4.3) for the JTA-200/2 and reference groups (individual change analysis without missing data imputation; Fig. 3a). The between-group difference in adjusted mean change from baseline was − 9.49 mm (95% CI: − 22.21, 3.23; *p* = 0.141); statistical superiority of the JTA-200/2 formulation over the reference was not demonstrated. When the primary endpoint was analyzed with missing data imputation, adjusted mean changes in WOMAC Pain Subscale Score from baseline were − 20.6 mm (SE: 4.3) and − 16.0 mm (4.3) for the JTA-200/2 and reference groups, with no statistically significant between-group difference (− 4.69 mm [95% CI: − 16.90, 7.52]; *p* = 0.447).

At Month 3, adjusted mean changes from baseline were − 26.8 mm (SE: 3.9) and − 15.1 mm (3.7) for the selected JTA-004 and reference groups (individual change analysis without missing data imputation), with no statistically significant (*p* > 0.025) between-group difference (− 11.63 mm [− 22.60, − 0.66]; *p* = 0.038) (Fig. 3b).

#### WOMAC Total score (final analysis)

At Month 3 and Month 6, adjusted mean changes from baseline in WOMAC Total Scores were − 22.3 mm (SE: 3.8) and − 23.7 mm (4.3) in the JTA-200/2 group, and − 19.4 mm (3.6) and − 16.5 mm (4.1) in the reference group (over time analysis; Table 4). There were no statistically significant differences between the JTA-200/2 and reference groups at any time point (*p* > 0.05).

**Table 4:** Difference in adjusted mean change from baseline in WOMAC Total Score (Full Analysis Set)

Difference between each JTA and the reference group in adjusted mean change from baseline in WOMAC Total Score	JTA-100/2*	JTA-200/2*	JTA-200/4*
**Month 3**			
**Change from Baseline**			
N	38	34	41
Adjusted Mean (SE)	−23.2 (3.9)	−22.3 (3.8)	− 28.9 (3.6)
**Difference between treatments (JTA-004 minus reference) in Change from Baseline**			
Adjusted Mean (SE)	−4.34 (5.44)	−2.94 (5.30)	−10.55 (5.05)
Adjusted CI (a)	−18.21, 9.54	−16.52, 10.65	−23.42, 2.32
p-value	0.878	0.972	0.143
**Month 6**			
**Change from Baseline**			
N	36	35	40
Adjusted Mean (SE)	−27.3 (4.2)	−23.7 (4.3)	−25.6 (4.0)
**Difference between treatments (JTA-004 minus reference) in Change from Baseline**			
Adjusted Mean (SE)	−11.49 (5.84)	−7.17 (5.96)	−10.11 (5.68)
Adjusted CI (a)	−26.29, 3.32	−22.28, 7.94	−24.47, 4.24
p-value	0.177	0.599	0.246

*CI* confidence interval, *JTA-100/2* group of patients receiving an injection of JTA-004 with 100 μg clonidine and 20 mg hyaluronic acid, *JTA-200/2* group of patients receiving an injection of JTA-004 with 200 μg clonidine and 20 mg hyaluronic acid, *JTA-200/4* group of patients receiving an injection of JTA-004 with 200 μg clonidine and 40 mg hyaluronic acid; N, total number of patients; SE, standard error; WOMAC, Western Ontario McMaster Universities. *N = 41 in each group

(a) Calculated using Dunnett-corrected t-value

Changes from baseline in WOMAC Total Score over time were evaluated using a Mixed-effect Model for Repeated Measurements with absolute change from baseline to the visit in WOMAC Total Score as response variable, treatment group and visit as factors, baseline WOMAC Total Score as covariate and treatment group-visit interaction

#### WOMAC physical function and stiffness subscale score (final analysis)

In the JTA-200/2 and reference groups, mean WOMAC Physical Function Subscale Scores at subsequent visits were lower than at baseline (over time analysis; Fig. 2b). At Month 3 and Month 6, adjusted mean changes from baseline in WOMAC Physical Function Subscale Score were − 23.8 mm (SE: 3.7) and − 26.1 mm (4.2) in the JTA-200/2 group, and − 17.7 mm (3.5) and − 16.1 mm (4.0) in the reference group, with no statistically significant between-group difference (p > 0.05).

In the JTA-200/2 and reference groups, mean WOMAC Stiffness Subscale Scores at subsequent visits were lower than at baseline (over time analysis). At Month 3 and Month 6, adjusted mean changes from baseline in WOMAC Stiffness Subscale Score were − 19.3 mm (SE: 4.5) and − 22.3 mm (4.5) in the JTA-200/2 group, and − 25.2 mm (4.3) and − 18.7 mm (4.3) in the reference group, with no statistically significant between-group difference (p > 0.05).

#### Well-being scores (final analysis)

Individual SF-12 Physical and Mental Component Summary Scores were highly variable in both the JTA-200/2 and reference groups (Additional file 2, Supplementary Tables 4 and 5). No conclusion could be drawn from these data.

#### Consumption of analgesics and NSAIDs (final analysis)

There were no consistent changes in consumption of analgesics and NSAIDs in either treatment group over time and no clinically relevant differences between treatment groups (Additional file 2, Supplementary Tables 6 and 7).

#### *Post-hoc* exploratory analyses

In *post-hoc* analyses, observed mean WOMAC Pain Subscale Scores at subsequent visits were lower than at baseline in both non-selected JTA groups (JTA-100/2 and JTA-200/4; over time analysis; Additional file 3, Supplementary Figs. 1a and 2a). The statistical significance criterion was only met for the JTA-200/4 group at Month 3 (individual change analysis; Fig. 3b). When analyzing the pooled JTA group and the reference group, changes in adjusted mean WOMAC Pain Subscale Score from baseline were − 27.2 mm (SE: 2.2) and − 16.4 mm (3.7) at Month 3, and − 26.1 mm (2.4) and − 15.6 mm (4.1) at Month 6, for the respective groups. Differences in adjusted mean changes from baseline between the pooled JTA and reference groups were − 10.79 mm (*p* = 0.014) at Month 3 and − 10.57 mm (*p* = 0.030) at Month 6, meeting the statistical significance criteria (individual change analysis; Fig. 3).

For the WOMAC Total Scores, *post-hoc* analyses in the non-selected JTA groups (JTA-100/2 and JTA-200/4) and in the pooled JTA group compared with the reference group showed no statistically significant between-group differences in adjusted mean change from baseline (over time analysis; Table 4, Additional file 2, Supplementary Table 8).

For the WOMAC Physical Function Subscale Score, *post-hoc* analyses in the non-selected JTA groups (JTA-100/2 and JTA-200/4) compared with the reference groups showed no statistically significant between-group differences in adjusted mean change from baseline (over time analysis; Additional file 3, Supplementary Figs. 1b and 2b). When analyzing the pooled JTA group and the reference group, adjusted mean changes from baseline in WOMAC Physical Function Subscale Score were − 27.0 mm (SE: 2.2) and − 18.0 mm (3.7) at Month 3, and − 26.7 mm (2.4) and − 17.3 mm (4.1) at Month 6, for the respective groups (data not shown). Differences in adjusted mean changes from baseline in WOMAC Physical Function Subscale Score between the pooled JTA and reference groups were − 8.97 mm (SE: 4.32) at Month 3 (*p* = 0.040) and − 9.40 mm (4.81) at Month 6 (*p* = 0.053), meeting the statistical significance criteria at Month 3 (individual change analysis; Additional file 3, Supplementary Fig. 3).

### Safety results

The mean follow-up duration for the 164 treated patients was 6.3 months (SD: 1.0). During the study, 116 (70.7%) patients experienced 292 AEs, with no significant differences between groups. Among these, 49 AEs were considered treatment-related by the investigator: 5 in 3 (7.3%) patients in the JTA-100/2, 12 in 8 (19.5%) patients in the JTA-200/2, 15 in 12 (29.3%) patients in the JTA-200/4, and 17 in 11 (26.8%) patients in the reference groups. Fewer treatment-related events were observed in the JTA-100/2 group (Table 5).

**Table 5:** Adverse events related to study treatment (Safety Set)

	JTA-100/2^a^		JTA-200/2^a^		JTA-200/4^a^		reference^a^	
	m	n (%)	m	n (%)	m	n (%)	m	n (%)
At least one AE related to study treatment	5	3 (7.3)	12	8 (19.5)	15	12 (29.3)	17	11 (26.8)
Musculoskeletal and Connective Tissue Disorders	1	1 (2.4)	1	1 (2.4)	3	3 (7.3)	6	5 (12.2)
Arthralgia	0	0 (0.0)	1	1 (2.4)	0	0 (0.0)	4	3 (7.3)
Osteoarthritis^b^	0	0 (0.0)	0	0 (0.0)	1	1 (2.4)	1	1 (2.4)
Joint Stiffness	1	1 (2.4)	0	0 (0.0)	0	0 (0.0)	0	0 (0.0)
Pain in Extremity	0	0 (0.0)	0	0 (0.0)	1	1 (2.4)	0	0 (0.0)
Plantar Fasciitis	0	0 (0.0)	0	0 (0.0)	0	0 (0.0)	1	1 (2.4)
Tendonitis	0	0 (0.0)	0	0 (0.0)	1	1 (2.4)	0	0 (0.0)
General Disorders and Administration Site Conditions	0	0 (0.0)	3	3 (7.3)	3	3 (7.3)	3	3 (7.3)
Injection Site Pain	0	0 (0.0)	0	0 (0.0)	1	1 (2.4)	2	2 (4.9)
Fatigue	0	0 (0.0)	0	0 (0.0)	1	1 (2.4)	1	1 (2.4)
Application Site Edema	0	0 (0.0)	1	1 (2.4)	0	0 (0.0)	0	0 (0.0)
Condition Aggravated	0	0 (0.0)	1	1 (2.4)	0	0 (0.0)	0	0 (0.0)
Influenza Like Illness	0	0 (0.0)	1	1 (2.4)	0	0 (0.0)	0	0 (0.0)
Thirst	0	0 (0.0)	0	0 (0.0)	1	1 (2.4)	0	0 (0.0)
Injury, Poisoning and Procedural Complications	0	0 (0.0)	1	1 (2.4)	3	3 (7.3)	1	1 (2.4)
Procedural Hypotension	0	0 (0.0)	0	0 (0.0)	3	3 (7.3)	0	0 (0.0)
Delayed Recovery from Anesthesia	0	0 (0.0)	1	1 (2.4)	0	0 (0.0)	0	0 (0.0)
Procedural Pain	0	0 (0.0)	0	0 (0.0)	0	0 (0.0)	1	1 (2.4)
Skin and Subcutaneous Tissue Disorders	1	1 (2.4)	1	1 (2.4)	3	3 (7.3)	0	0 (0.0)
Eczema	0	0 (0.0)	0	0 (0.0)	1	1 (2.4)	0	0 (0.0)
Erythema	0	0 (0.0)	1	1 (2.4)	0	0 (0.0)	0	0 (0.0)
Hyperkeratosis	1	1 (2.4)	0	0 (0.0)	0	0 (0.0)	0	0 (0.0)
Skin Irritation	0	0 (0.0)	0	0 (0.0)	1	1 (2.4)	0	0 (0.0)
Skin Lesion	0	0 (0.0)	0	0 (0.0)	1	1 (2.4)	0	0 (0.0)
Investigations	2	1 (2.4)	1	1 (2.4)	0	0 (0.0)	2	2 (4.9)
Blood Creatine Phosphokinase Increased	0	0 (0.0)	0	0 (0.0)	0	0 (0.0)	2	2 (4.9)
Amylase Increased	1	1 (2.4)	0	0 (0.0)	0	0 (0.0)	0	0 (0.0)
Blood Pressure Decreased	0	0 (0.0)	1	1 (2.4)	0	0 (0.0)	0	0 (0.0)
Blood Triglycerides Increased	1	1 (2.4)	0	0 (0.0)	0	0 (0.0)	0	0 (0.0)
Gastrointestinal Disorders	0	0 (0.0)	1	1 (2.4)	1	1 (2.4)	1	1 (2.4)
Abdominal Pain	0	0 (0.0)	0	0 (0.0)	1	1 (2.4)	0	0 (0.0)
Diarrhea	0	0 (0.0)	1	1 (2.4)	0	0 (0.0)	0	0 (0.0)
Melaena	0	0 (0.0)	0	0 (0.0)	0	0 (0.0)	1	1 (2.4)
Cardiac Disorders	0	0 (0.0)	1	1 (2.4)	1	1 (2.4)	0	0 (0.0)
Supraventricular Extrasystoles	0	0 (0.0)	0	0 (0.0)	1	1 (2.4)	0	0 (0.0)
Tachycardia	0	0 (0.0)	1	1 (2.4)	0	0 (0.0)	0	0 (0.0)
Surgical and Medical Procedures	1	1 (2.4)	0	0 (0.0)	0	0 (0.0)	1	1 (2.4)
Joint Injection	1	1 (2.4)	0	0 (0.0)	0	0 (0.0)	1	1 (2.4)
Ear and Labyrinth Disorders	0	0 (0.0)	0	0 (0.0)	0	0 (0.0)	1	1 (2.4)
Vertigo	0	0 (0.0)	0	0 (0.0)	0	0 (0.0)	1	1 (2.4)
Infections and Infestations	0	0 (0.0)	3	1 (2.4)	0	0 (0.0)	0	0 (0.0)
Osteomyelitis Acute	0	0 (0.0)	1	1 (2.4)	0	0 (0.0)	0	0 (0.0)
Osteomyelitis Chronic	0	0 (0.0)	2	1 (2.4)	0	0 (0.0)	0	0 (0.0)
Nervous System Disorders	0	0 (0.0)	0	0 (0.0)	0	0 (0.0)	1	1 (2.4)
Headache	0	0 (0.0)	0	0 (0.0)	0	0 (0.0)	1	1 (2.4)
Psychiatric Disorders	0	0 (0.0)	0	0 (0.0)	0	0 (0.0)	1	1 (2.4)
Major Depression	0	0 (0.0)	0	0 (0.0)	0	0 (0.0)	1	1 (2.4)
Vascular Disorders	0	0 (0.0)	0	0 (0.0)	1	1 (2.4)	0	0 (0.0)
Hypotension	0	0 (0.0)	0	0 (0.0)	1	1 (2.4)	0	0 (0.0)

AE, adverse event; JTA-100/2, group of patients receiving an injection of JTA-004 with 100 μg clonidine and 20 mg hyaluronic acid; JTA-200/2, group of patients receiving an injection of JTA-004 with 200 μg clonidine and 20 mg hyaluronic acid; JTA-200/4, group of patients receiving an injection of JTA-004 with 200 μg clonidine and 40 mg hyaluronic acid; n, number of patients with at least one serious AE; N, total number of patients; %, (n row / N group) × 100; m, number of serious adverse events; reference, group of patients receiving an injection of the reference treatment (hylan G-F 20). ^a^N = 41 in each group. ^b^Knee gonarthrosis in one patient in the JTA-200/4 group and arthrosis crisis in one patient in the reference group

Moreover, 36 AEs were considered related to study procedures by the investigator: 4 in 3 (7.3%) patients in the JTA-100/2, 10 in 6 (14.6%) patients in the JTA-200/2, 12 in 9 (22.0%) patients in the JTA-200/4, and 10 in 8 (19.5%) patients in the reference groups (Additional file 2, Supplementary Table 9). Again, fewer procedure-related events were observed in the JTA-100/2 group.

The most frequently reported study treatment- or procedure-related AEs across all groups were arthralgia, injection site pain, and hypotension (4 [9.8%] patients in the JTA-200/4, 2 [4.9%] patients in the JTA-200/2, and no patient in the JTA-100/2 and reference groups had mild and short-lasting hypotension after injection).

Eight patients experienced 11 SAEs: 4 in 4 (9.8%) patients in the JTA-100/2, 5 in 2 (4.9%) patients in the JTA-200/2, 1 (2.4% of patients) in the JTA-200/4, and 1 (2.4% of patients) in the reference groups. No SAEs were reported by more than one patient (Table 6).

**Table 6:** Serious adverse events (Safety Set)

	JTA-100/2^a^		JTA-200/2^a^		JTA-200/4^a^		reference^a^	
	m	n (%)	m	n (%)	m	n (%)	m	n (%)
At least one serious AE	4	4 (9.8)	5	2 (4.9)	1	1 (2.4)	1	1 (2.4)
Gastrointestinal Disorders	2	2 (4.9)	1	1 (2.4)	0	0 (0.0)	0	0 (0.0)
Barrett’s Esophagus	1	1 (2.4)	0	0 (0.0)	0	0 (0.0)	0	0 (0.0)
Diarrhea	1	1 (2.4)	0	0 (0.0)	0	0 (0.0)	0	0 (0.0)
Hernial Eventration	0	0 (0.0)	1	1 (2.4)	0	0 (0.0)	0	0 (0.0)
Infections and Infestations	0	0 (0.0)	3	1 (2.4)	1	1 (2.4)	0	0 (0.0)
Osteomyelitis Acute	0	0 (0.0)	1	1 (2.4)	0	0 (0.0)	0	0 (0.0)
Osteomyelitis Chronic	0	0 (0.0)	2	1 (2.4)	0	0 (0.0)	0	0 (0.0)
Pneumonia	0	0 (0.0)	0	0 (0.0)	1	1 (2.4)	0	0 (0.0)
Injury, Poisoning and Procedural Complications	0	0 (0.0)	0	0 (0.0)	0	0 (0.0)	1	1 (2.4)
Alcohol Poisoning	0	0 (0.0)	0	0 (0.0)	0	0 (0.0)	1	1 (2.4)
Musculoskeletal and Connective Tissue Disorders	1	1 (2.4)	0	0 (0.0)	0	0 (0.0)	0	0 (0.0)
Arthralgia	1	1 (2.4)	0	0 (0.0)	0	0 (0.0)	0	0 (0.0)
Neoplasms Benign, Malignant and Unspecified (Incl Cysts and Polyps)	0	0 (0.0)	1	1 (2.4)	0	0 (0.0)	0	0 (0.0)
Squamous Cell Carcinoma of Lung	0	0 (0.0)	1	1 (2.4)	0	0 (0.0)	0	0 (0.0)
Reproductive System and Breast Disorders	1	1 (2.4)	0	0 (0.0)	0	0 (0.0)	0	0 (0.0)
Rectocele	1	1 (2.4)	0	0 (0.0)	0	0 (0.0)	0	0 (0.0)

*AE* adverse event, *m* number of serious adverse events, *JTA-100/2* group of patients receiving an injection of JTA-004 with 100 μg clonidine and 20 mg hyaluronic acid, *JTA-200/2* group of patients receiving an injection of JTA-004 with 200 μg clonidine and 20 mg hyaluronic acid, *JTA-200/4* group of patients receiving an injection of JTA-004 with 200 μg clonidine and 40 mg hyaluronic acid, *n* number of patients with at least one serious AE, *N* total number of patients; reference, group of patients receiving an injection of the reference treatment (hylan G-F 20); %, (n row / N group) × 100. ^a^N = 41 in each group

One patient in the JTA-200/2 group experienced 3 SAEs considered as possibly related to study treatment or procedures: 1 acute osteomyelitis event and 2 chronic osteomyelitis events at the same location. Acute osteomyelitis was reported as a Suspected Unexpected Serious Adverse Reaction (SUSAR) by the sponsor as precautionary measure. Upon complete case review, the event was assessed as not study treatment- or procedure-related. More details are given in Additional file 1, Supplementary Text 2.

Two patients experienced 2 AEs leading to study withdrawal: 1 in the JTA-100/2 group (arthralgia) and 1 in the JTA-200/2 group (acute osteomyelitis; SUSAR). No deaths were reported.

## Discussion

This study aimed to select the most effective formulation of a new enhanced protein solution (JTA-004) for knee OA treatment and to compare its efficacy and safety with a reference treatment (hylan G-F 20) during 6 months.

The JTA-004 formulation selected for final analyses contained 200 μg clonidine and 20 mg HA in 2 ml (JTA-200/2). Its statistical superiority over the reference treatment could not be demonstrated by the difference in adjusted mean changes in WOMAC Pain Subscale Score from baseline at 6 months post-injection, although a clinically relevant difference between groups was observed (> 10 mm, the MCID). This non-significant result for the primary endpoint was probably due to the lack of study power, rather than lack of effect, due to the larger variation of the primary endpoint estimates (observed SD: 25–30 mm) than anticipated based on the literature (expected SD: 10.5 mm). Although the selected JTA-004 formulation induced larger improvements than the reference in WOMAC Pain Subscale, Physical Function Subscale, and Total Scores at all timepoints, no differences met the statistical significance criteria. *Post-hoc* analyses of the non-selected JTA-004 formulations (JTA-100/2 and JTA-200/4) provided results similar to the selected formulation, and no clear benefit of one formulation over another could be evidenced. In other *post-hoc* analyses, improvements in WOMAC Pain Subscale Scores compared with baseline were larger in the pooled patients who received any of the three JTA-004 formulations than patients receiving the reference treatment. Here, statistical significance criteria were met, and differences were clinically important (> 10 mm, the MCID) at 3 and 6 months post-injection. Improvements in the WOMAC Physical Function Subscale from baseline were also statistically significantly larger in the pooled patients who received JTA-004 than the reference at 3 months post-injection.

While our primary efficacy analysis was statistically inconclusive, the larger pain and function improvements observed with JTA-004 compared with the reference are encouraging since injections of HA or its derivatives were shown to result in pain relief, and joint function and quality of life improvements in knee OA patients [28, 31–35], leading to the introduction of intra-articular HA injections in international recommendations [36]. However, other studies have shown that pain and function improvements post-HA injections were similar or only slightly higher than with saline placebo, highlighting the importance of the placebo effect with intra-articular injections [18, 37–39]. In our study, the potentially larger effect observed with JTA-004 compared with the reference may be explained by the fact that besides the natural HA polysaccharide obtained by bacterial fermentation, JTA-004 also contains active substances with jellification and anti-inflammatory properties (human plasma protein solution). JTA-004 jellifies through the coagulation cascade and forms a clotting gel, resulting in a tridimensional network entrapping HA fibers, plasma proteins, and synovial proteins of the patient [17]. This formulation with the resulting clotting gel offers the potential for a better lubrication and protection of the cartilage with a prolonged effect (unpublished data).

Other injectable medications causing regenerative changes in tissue structure and reducing OA symptoms have been developed. Blood derivatives, especially autologous PRP intra-articular injections stimulating the cartilage healing process and improving the damage caused by articular disease, were shown to have a superior effect than HA for knee OA treatment [10, 12]. Two randomized studies have shown that combinations of PRP and HA improved arthralgia and increased physical function compared with PRP or HA alone [40, 41]. Intra-articular Plasma Rich in Growth Factor injections are also under investigation [42]. Although we did not compare these treatments with JTA-004, the potentially larger improvement induced by our enhanced protein solution compared with the reference may indicate that JTA-004 could be an effective treatment option for knee OA patients. Other intra-articular treatments are currently evaluated but were not compared with HA injections. They include intra-articular triamcinolone acetonide extended-release injections, approved by the United States Food and Drug Administration to treat knee OA [43], and intra-articular capsaicin, which has analgesic properties and induced significant improvement in pain compared with placebo in knee OA patients in a phase II study [44].

In our study, all evaluated JTA-004 formulations showed a clinically acceptable safety profile. Four patients in the JTA-200/4 and two patients in the JTA-200/2 groups had mild and short-lasting post-injection hypotension, which may be caused by the clonidine (anti-hypertensive medication) contained in JTA-004 or by a vasovagal episode. There were fewer treatment-related events observed in the JTA-100/2 group, notably no cases of post-injection hypotension.

The limitations of this double-blind study included the larger inter-patient variability than anticipated for all assessed clinical endpoints, shown by the SD amplitudes, and the lack of study power due to the fact that the IDMC recommendation to increase the sample size to 76 participants per group was not followed at the time of the interim analysis. Other limitations were the fact that the study was only conducted in one country and the differences in terms of preparation between the JTA-004 formulations (kits containing vials of freeze-dried JTA-004 and resuspension solution) and the reference treatment (ready-to-use syringe). A further limitation was the fact that patients did not have to stop medications before measuring the outcomes at the various study visits. In addition, while both men and women were included in the study, no subanalyses based on sex or gender were performed. A further drawback was the absence of placebo-receiving control group; nevertheless, this was accounted for by our choice of reference, which had shown superiority over placebo [18–20, 35].

The present study showed a clinically acceptable safety profile of all evaluated JTA-004 formulations and provided preliminary evidence of the efficacy of JTA-004 for the treatment of symptomatic knee OA. Our results did not indicate a statistically significant benefit of one specific JTA-004 formulation with respect to the reference treatment. Nevertheless, the statistically significant superiority of the pooled JTA group versus the reference group in the *post-hoc* analyses indicates a potentially clinically relevant impact of JTA-004. The JTA-100/2 formulation showed a more favorable safety profile with comparable efficacy and was selected for Phase III studies.

## Conclusions

This study provided preliminary evidence of the safety of intra-articular injections of our enhanced protein solution JTA-004 for the treatment of symptomatic knee OA. While we did not demonstrate a superior efficacy of the selected JTA-004 formulation over the reference treatment, *post-hoc* analyses on pooled data from all formulations showed statistically significantly larger improvements in WOMAC Pain Subscale Scores for JTA-004 than the reference at Month 6 and Month 3. A phase III randomized controlled trial with a larger sample size is needed to evaluate whether JTA-004 is effective and could be an alternative minimally invasive viscosupplement therapeutic option to control pain and delay joint replacement surgery in patients with knee OA.

## Supplementary Information

**Additional file 1: Supplementary Text 1.** Exclusion Criteria**. Supplementary Text 2.** Narrative of the adverse event considered as a Suspected Unexpected Serious Adverse Reaction (SUSAR).

**Additional file 2: Supplementary Table 1.** List of primary, secondary and exploratory predefined efficacy endpoints in the second study stage**. Supplementary Table 2.** Difference between each JTA group and the reference group in adjusted mean change from baseline in WOMAC Total Score (interim analysis; Full Analysis Set)**. Supplementary Table 3.** Difference between each JTA group and the reference group in adjusted mean change from baseline in WOMAC Physical Function Subscale Score (interim analysis; Full Analysis Set)**. Supplementary Table 4.** Difference between each JTA-004 treatment group and the reference group in adjusted mean change from baseline in SF-12 Well-Being Scores (Physical Component Summary Score) (Full Analysis Set)**. Supplementary Table 5.** Difference between each JTA-004 treatment group and the reference group in adjusted mean change from baseline in SF-12 Well-Being Scores (Mental Component Summary Score) (Full Analysis Set)**. Supplementary Table 6.** Consumption of analgesics - Values by visit and absolute change from baseline (Full Analysis Set)**. Supplementary Table 7.** Consumption of NSAIDs - Values by visit and absolute change from baseline (Full Analysis Set)**. Supplementary Table 8.** Difference between the pooled JTA group and the reference group in adjusted mean change from baseline in WOMAC Total Score over time (Full Analysis Set)**. Supplementary Table 9.** Adverse events related to study procedures (Safety Set).

**Additional file 3: Supplementary Fig. 1**. Change from baseline in WOMAC (A) Pain Subscale and (B) Physical Function Subscale Score over time in the JTA-100/2 group and in the reference group (Full Analysis Set)**. Supplementary Fig. 2.** Change from baseline in WOMAC (A) Pain Subscale and (B) Physical Function Subscale Score over time in the JTA-200/4 group and in the reference group (Full Analysis Set)**. Supplementary Fig. 3.** Difference between the pooled JTA-004 group and the reference group in adjusted mean change from baseline in WOMAC Physical Function Subscale Score at Month 3 and Month 6 (Full Analysis Set).

## Data Availability

The datasets used and/or analyzed during the current study are available from the corresponding author on reasonable request.

## Abbreviations

AE: Adverse event
ANCOVA: Analysis of covariance
BMI: Body mass index
CI: Confidence interval
FAS: Full Analysis Set
HA: Hyaluronic acid
IDMC: Independent Data Monitoring Committee
JTA-004: enhanced protein solution supplemented with hyaluronic acid and clonidine
JTA-100/2 group: group of patients receiving an injection of JTA-004 with 100 μg clonidine and 20 mg hyaluronic acid
JTA-200/2 group: group of patients receiving an injection of JTA-004 with 200 μg clonidine and 20 mg hyaluronic acid
JTA-200/4 group: group of patients receiving an injection of JTA-004 with 200 μg clonidine and 40 mg hyaluronic acid
MCID: Minimal clinically important difference
NSAID: Non-steroidal anti-inflammatory drug
OA: Osteoarthritis
PRP: Platelet-rich plasma
SAE: Serious adverse event
SD: Standard deviation
SE: Standard error
SF-12: 12-item Short Form Health Survey
SUSAR: Suspected unexpected serious adverse reaction
WOMAC: Western Ontario McMaster Universities

## References

[1] Hochberg MC, Yerges-Armstrong L, Yau M, Mitchell BD (2013). Genetic epidemiology of osteoarthritis: recent developments and future directions. Curr Opin Rheumatol.

[2] Ayhan E, Kesmezacar H, Akgun I (2014). Intraarticular injections (corticosteroid, hyaluronic acid, platelet rich plasma) for the knee osteoarthritis. World J Orthop.

[3] Wang F, He X (2015). Intra-articular hyaluronic acid and corticosteroids in the treatment of knee osteoarthritis: a meta-analysis. Exp Ther Med.

[4] Jones IA, Togashi R, Wilson ML, Heckmann N, Vangsness CT (2019). Intra-articular treatment options for knee osteoarthritis. Nat Rev Rheumatol.

[5] Bellamy N, Campbell J, Robinson V, Gee T, Bourne R, Wells G. Intraarticular corticosteroid for treatment of osteoarthritis of the knee. Cochrane Database Syst Rev. 2006:CD005328. 10.1002/14651858.CD005328.pub2. 10.1002/14651858.CD005328.pub2 16625636

[6] Nicholls MA, Fierlinger A, Niazi F, Bhandari M (2017). The disease-modifying effects of hyaluronan in the osteoarthritic disease state. Clin Med Insights Arthritis Musculoskelet Disord.

[7] Salmon JH, Rat AC, Charlot-Lambrecht I, Eschard JP, Jolly D, Fautrel B (2018). Cost effectiveness of intra-articular hyaluronic acid and disease-modifying drugs in knee osteoarthritis. Pharmacoeconomics..

[8] Glynn LG, Mustafa A, Casey M, Krawczyk J, Blom J, Galvin R, Hannigan A, Dunne CP, Murphy AW, Mallen C (2018). Platelet-rich plasma (PRP) therapy for knee arthritis: a feasibility study in primary care. Pilot Feasibility Stud.

[9] Dai WL, Zhou AG, Zhang H, Zhang J (2017). Efficacy of platelet-rich plasma in the treatment of knee osteoarthritis: A meta-analysis of randomized controlled trials. Arthroscopy.

[10] Laudy AB, Bakker EW, Rekers M, Moen MH (2015). Efficacy of platelet-rich plasma injections in osteoarthritis of the knee: a systematic review and meta-analysis. Br J Sports Med.

[11] Shen L, Yuan T, Chen S, Xie X, Zhang C (2017). The temporal effect of platelet-rich plasma on pain and physical function in the treatment of knee osteoarthritis: systematic review and meta-analysis of randomized controlled trials. J Orthop Surg Res.

[12] Milants C, Bruyere O, Kaux JF (2017). Responders to platelet-rich plasma in osteoarthritis: a technical analysis. Biomed Res Int.

[13] Milants C, Bruyere O, Kaux JF (2018). Response to: comment on "responders to platelet-rich plasma in osteoarthritis: a technical analysis". Biomed Res Int.

[14] Fuggle NR, Cooper C, Oreffo ROC, Price AJ, Kaux JF, Maheu E, Cutolo M, Honvo G, Conaghan PG, Berenbaum F (2020). Alternative and complementary therapies in osteoarthritis and cartilage repair. Aging Clin Exp Res.

[15] Gentili M, Juhel A, Bonnet F (1996). Peripheral analgesic effect of intra-articular clonidine. Pain..

[16] Sun R, Zhao W, Hao Q, Tian H, Tian J, Li L, Jia W, Yang K (2014). Intra-articular clonidine for post-operative analgesia following arthroscopic knee surgery: a systematic review and meta-analysis. Knee Surg Sports Traumatol Arthrosc.

[17] Martin-Alarcon L, Schmidt TA (2016). Rheological effects of macromolecular interactions in synovial fluid. Biorheology..

[18] Chevalier X, Jerosch J, Goupille P, van Dijk N, Luyten FP, Scott DL, Bailleul F, Pavelka K (2010). Single, intra-articular treatment with 6 ml hylan G-F 20 in patients with symptomatic primary osteoarthritis of the knee: a randomised, multicentre, double-blind, placebo controlled trial. Ann Rheum Dis.

[19] Huskin JP, Vandekerckhove B, Delince P, Verdonk R, Dubuc JE, Willems S, Hardy P, Blanco FJ, Charrois O, Handelberg F (2008). Multicentre, prospective, open study to evaluate the safety and efficacy of hylan G-F 20 in knee osteoarthritis subjects presenting with pain following arthroscopic meniscectomy. Knee Surg Sports Traumatol Arthrosc.

[20] Raman R, Dutta A, Day N, Sharma HK, Shaw CJ, Johnson GV (2008). Efficacy of Hylan G-F 20 and sodium hyaluronate in the treatment of osteoarthritis of the knee -- a prospective randomized clinical trial. Knee..

[21] Braun HJ, Gold GE (2012). Diagnosis of osteoarthritis: imaging. Bone..

[22] Kohn MD, Sassoon AA, Fernando ND (2016). Classifications in brief: Kellgren-Lawrence classification of osteoarthritis. Clin Orthop Relat Res.

[23] Jackson DW, Evans NA, Thomas BM (2002). Accuracy of needle placement into the intra-articular space of the knee. J Bone Joint Surg Am.

[24] Bellamy N (2005). The WOMAC knee and hip osteoarthritis indices: development, validation, globalization and influence on the development of the AUSCAN hand osteoarthritis indices. Clin Exp Rheumatol.

[25] Ehrich EW, Davies GM, Watson DJ, Bolognese JA, Seidenberg BC, Bellamy N (2000). Minimal perceptible clinical improvement with the Western Ontario and McMaster universities osteoarthritis index questionnaire and global assessments in patients with osteoarthritis. J Rheumatol.

[26] Angst F, Aeschlimann A, Stucki G (2001). Smallest detectable and minimal clinically important differences of rehabilitation intervention with their implications for required sample sizes using WOMAC and SF-36 quality of life measurement instruments in patients with osteoarthritis of the lower extremities. Arthritis Rheum.

[27] Bellamy N, Carr A, Dougados M, Shea B, Wells G (2001). Towards a definition of "difference" in osteoarthritis. J Rheumatol.

[28] Borras-Verdera A, Calcedo-Bernal V, Ojeda-Levenfeld J, Clavel-Sainz C (2012). Efficacy and safety of a single intra-articular injection of 2% hyaluronic acid plus mannitol in knee osteoarthritis over a 6-month period. Rev Esp Cir Ortop Traumatol.

[29] Pavelka K, Uebelhart D (2011). Efficacy evaluation of highly purified intra-articular hyaluronic acid (Sinovial(®)) vs hylan G-F20 (Synvisc(®)) in the treatment of symptomatic knee osteoarthritis. A double-blind, controlled, randomized, parallel-group non-inferiority study. Osteoarthr Cartil.

[30] Koenig F, Brannath W, Bretz F, Posch M (2008). Adaptive Dunnett tests for treatment selection. Stat Med.

[31] Altman RD, Rosen JE, Bloch DA, Hatoum HT, Korner P (2009). A double-blind, randomized, saline-controlled study of the efficacy and safety of EUFLEXXA for treatment of painful osteoarthritis of the knee, with an open-label safety extension (the FLEXX trial). Semin Arthritis Rheum.

[32] Berenbaum F, Grifka J, Cazzaniga S, D'Amato M, Giacovelli G, Chevalier X, Rannou F, Rovati LC, Maheu E (2012). A randomised, double-blind, controlled trial comparing two intra-articular hyaluronic acid preparations differing by their molecular weight in symptomatic knee osteoarthritis. Ann Rheum Dis.

[33] Juni P, Reichenbach S, Trelle S, Tschannen B, Wandel S, Jordi B, Zullig M, Guetg R, Hauselmann HJ, Schwarz H (2007). Efficacy and safety of intraarticular hylan or hyaluronic acids for osteoarthritis of the knee: a randomized controlled trial. Arthritis Rheum.

[34] Petrella RJ (2005). Hyaluronic acid for the treatment of knee osteoarthritis: long-term outcomes from a naturalistic primary care experience. Am J Phys Med Rehabil.

[35] Vannabouathong C, Bhandari M, Bedi A, Khanna V, Yung P, Shetty V, Khan M (2018). Nonoperative treatments for knee osteoarthritis: an evaluation of treatment characteristics and the intra-articular placebo effect: a systematic review. JBJS Rev.

[36] Bruyere O, Honvo G, Veronese N, Arden NK, Branco J, Curtis EM, et al. An updated algorithm recommendation for the management of knee osteoarthritis from the European Society for Clinical and Economic Aspects of Osteoporosis, Osteoarthritis and Musculoskeletal Diseases (ESCEO). Semin Arthritis Rheum. 2019. 10.1016/j.semarthrit.2019.04.008. 10.1016/j.semarthrit.2019.04.008 31126594

[37] Lundsgaard C, Dufour N, Fallentin E, Winkel P, Gluud C (2008). Intra-articular sodium hyaluronate 2 mL versus physiological saline 20 mL versus physiological saline 2 mL for painful knee osteoarthritis: a randomized clinical trial. Scand J Rheumatol.

[38] Bihlet AR, Byrjalsen I, Bay-Jensen AC, Andersen JR, Christiansen C, Riis BJ, Valter I, Karsdal MA, Hochberg MC (2018). Identification of pain categories associated with change in pain in patients receiving placebo: data from two phase 3 randomized clinical trials in symptomatic knee osteoarthritis. BMC Musculoskelet Disord.

[39] Zhang W, Robertson J, Jones AC, Dieppe PA, Doherty M (2008). The placebo effect and its determinants in osteoarthritis: meta-analysis of randomised controlled trials. Ann Rheum Dis.

[40] Yu W, Xu P, Huang G, Liu L (2018). Clinical therapy of hyaluronic acid combined with platelet-rich plasma for the treatment of knee osteoarthritis. Exp Ther Med.

[41] Lana JF, Weglein A, Sampson SE, Vicente EF, Huber SC, Souza CV, Ambach MA, Vincent H, Urban-Paffaro A, Onodera CM (2016). Randomized controlled trial comparing hyaluronic acid, platelet-rich plasma and the combination of both in the treatment of mild and moderate osteoarthritis of the knee. J Stem Cells Regen Med.

[42] Raeissadat SA, Rayegani SM, Ahangar AG, Abadi PH, Mojgani P, Ahangar OG (2017). Efficacy of intra-articular injection of a newly developed plasma rich in growth factor (PRGF) versus hyaluronic acid on pain and function of patients with knee osteoarthritis: a single-blinded randomized clinical trial. Clin Med Insights Arthritis Musculoskelet Disord.

[43] Paik J, Duggan ST, Keam SJ (2019). Triamcinolone acetonide extended-release: a review in osteoarthritis pain of the knee. Drugs..

[44] Stevens RM, Ervin J, Nezzer J, Nieves Y, Guedes K, Burges R, Hanson PD, Campbell JN (2019). Randomized, double-blind, placebo-controlled trial of intraarticular trans-capsaicin for pain associated with osteoarthritis of the knee. Arthritis Rheum.

