# Supplementary material for: Safety and efficacy of a single intra-articular injection of a novel enhanced protein solution (JTA-004) compared to hylan G-F 20 in symptomatic knee osteoarthritis: a randomized, double-blind, controlled phase II/III study
Source: BMC Musculoskelet Disord. 2021 Oct 19;22:888. doi: 10.1186/s12891-021-04750-3 (PMC8527807; doi:10.1186/s12891-021-04750-3)
Supplement: Supplementary file 1 — Additional file 1: Supplementary Text 1. Exclusion Criteria. Supplementary Text 2. Narrative of the adverse event considered as a Suspected Unexpected Serious Adverse Reaction (SUSAR). [file 12891_2021_4750_MOESM1_ESM.docx]

**Additional file 1. Supplementary text**

**Supplementary Text 1.** Exclusion Criteria

Current symptoms and/or signs related to the disease under study

- Isolated symptomatic femoropatellar osteoarthritis of the target knee
- History of trauma or surgery or arthroscopy at the target knee within 6 months before inclusion
- Concomitant inflammatory disease or other condition affecting the joints (e.g., rheumatoid arthritis, septic arthritis, inflammatory joint disease, metabolic bone disease, psoriasis, gout, microcrystalline arthropathies/chondrocalcinosis, Paget’s disease)
- Any musculoskeletal condition (such as hip osteoarthritis, amputation, neurologic disorder) that would impede measurement of efficacy at target knee
- Target knee prosthesis planned within 12 months after the Screening Visit

Current or previous diagnoses, signs and/or symptoms

- Uncontrolled diabetes mellitus, end-stage hepatic or renal disease documented in the patient’s file
- Current (or within the last 5 years prior to entering the study) history of solid or hematological neoplasia or bone marrow transplantation (except for basal cell carcinoma and completely excised squamous cell carcinoma)
- Other severe acute or chronic medical or psychiatric conditions or pre-dispositions or laboratory abnormalities, as judged by the Investigator
- Current or past history of coagulation disorders, as judged by the Investigator
- Hypersensitivity to any components of hyaluronic acid-based injection products
- History of hypersensitivity to human biological material including blood and blood derived products, potential excipients and residues from manufacturing process, documented clinically or by laboratory tests
- Hypersensitivity to avian proteins
- Life expectancy less than 6 months

Current or previous treatment

- Participation in another clinical study within 6 months prior to Screening
- Patients previously treated with JTA-004
- Treatment:

- Within 6 months prior to Screening: intra-articular hyaluronic acid injection at the target knee

- Within 2 months prior to Screening: intra-articular glucocorticoids at the target knee

- Current chemo-, radio- or immuno-cancer-therapy or immunosuppressive therapy
- Current anti-hypertensive medication the effects of which are known to be potentiated by a single dose of clonidine
- Current (or within 6 months prior to Screening) illicit drug abuse

Safety aspects concerning female subjects of childbearing potential

- Females who are pregnant, lactating or women with childbearing potential (last menstrual bleeding less than 12 months ago) unwilling to use medically acceptable contraception, or women with childbearing potential unwilling to perform a pregnancy test before administration of study treatment.

**Supplementary Text 2.** Narrative of the adverse event considered as a Suspected Unexpected Serious Adverse Reaction (SUSAR)

The patient was a 65-year-old male randomized to the JTA-200/2 group. Relevant medical history included gonarthrosis, pain in the left thigh since November 2016, malaria, and atrial fibrillation. On 12 January 2017, the study treatment was administered in the patient's left knee. On 06 February 2017, the patient was hospitalized for acute osteomyelitis. During hospitalization, a computerized tomography (CT) scan revealed an osteolytic lesion of the femoral diaphysis with soft tissue infiltration, magnetic resonance imaging indicated a suspicion of a malignant lesion and ultrasound examination showed acute osteomyelitis. Bone biopsy was negative for malignant cells and revealed acute osteomyelitis and signs of chronic inflammation with destruction of bone characterized by the presence of osteoclasts. The event was assessed as serious and severe in intensity by the Investigator. The event was assessed as possibly related to the trial medication and study procedure by the Investigator. This event was reported as a SUSAR by the Sponsor as a precautionary measure, but upon complete review of the case, acute osteomyelitis was assessed as not related to trial medication or study procedure by the Sponsor. On 07 February 2017, the patient was discontinued from the study. Results of the microbiological examination revealed the presence of *Staphylococcus aureus* infection and the patient was diagnosed with chronic osteomyelitis. The event was treated by drainage and antibiotic therapy (flucloxacillin intravenously then orally). On 01 March 2017, the patient had an intramedullary curettage and bore of the left femur and had been prescribed antibiotic therapy. However, during the postoperative recovery period the patient developed a fistula at the surgery site with purulent discharge. On 05 May 2017, the patient was hospitalized due to a relapse of chronic osteomyelitis and a fistula surgery was performed. Due to the persistence of an inflammatory syndrome, the patient had an intramedullary curettage and bore of the left femur on 09 May 2017. A CT scan of the femoral bone performed on 17 October 2017 confirmed that the bone defect was persistent and that it extended about 2 cm in height between the middle and the lower layer of the left femoral bone (medial cortical).
