# Supplementary material for: Safety and efficacy of a single intra-articular injection of a novel enhanced protein solution (JTA-004) compared to hylan G-F 20 in symptomatic knee osteoarthritis: a randomized, double-blind, controlled phase II/III study
Source: BMC Musculoskelet Disord. 2021 Oct 19;22:888. doi: 10.1186/s12891-021-04750-3 (PMC8527807; doi:10.1186/s12891-021-04750-3)
Supplement: Supplementary file 2 — Additional file 2: Supplementary Table 1. List of primary, secondary and exploratory predefined efficacy endpoints in the second study stage. Supplementary Table 2. Difference between each JTA group and the reference group in adjusted mean change from baseline in WOMAC Total Score (interim analysis; Full Analysis Set). Supplementary Table 3. Difference between each JTA group and the reference group in adjusted mean change from baseline in WOMAC Physical Function Subscale Score (interim analysis; Full Analysis Set). Supplementary Table 4. Difference between each JTA-004 treatment group and the reference group in adjusted mean change from baseline in SF-12 Well-Being Scores (Physical Component Summary Score) (Full Analysis Set). Supplementary Table 5. Difference between each JTA-004 treatment group and the reference group in adjusted mean change from baseline in SF-12 Well-Being Scores (Mental Component Summary Score) (Full Analysis Set). Supplementary Table 6. Consumption of analgesics - Values by visit and absolute change from baseline (Full Analysis Set). Supplementary Table 7. Consumption of NSAIDs - Values by visit and absolute change from baseline (Full Analysis Set). Supplementary Table 8. Difference between the pooled JTA group and the reference group in adjusted mean change from baseline in WOMAC Total Score over time (Full Analysis Set). Supplementary Table 9. Adverse events related to study procedures (Safety Set). [file 12891_2021_4750_MOESM2_ESM.docx]

**Additional file 2. Supplementary tables**

**Supplementary Table 1.** List of primary, secondary and exploratory predefined efficacy endpoints in the second study stage

|  | **Month 3** | **Month 6** | **Over time** |
| --- | --- | --- | --- |
| WOMAC Pain Subscale | Secondary | Primary | Exploratory |
| WOMAC Total Score |  | Exploratory | Secondary |
| WOMAC Physical Function Subscale | - | Exploratory | Exploratory |
| WOMAC Stiffness Subscale | - | Exploratory | Exploratory |
| Well-being Scores | - | Exploratory | Exploratory |
| Consumption of analgesics | - | Exploratory | Exploratory |
| Consumption of NSAIDs | - | Exploratory | Exploratory |

NSAID, nonsteroidal anti-inflammatory drug; WOMAC, Western Ontario McMaster Universities.

**Supplementary Table 2.** Difference between each JTA group and the reference group in adjusted mean change from baseline in WOMAC Total Score (interim analysis; Full Analysis Set)

|  | **JTA-100/2** | **JTA-200/2** | **JTA-200/4** |
| --- | --- | --- | --- |
| **Month 3** |  |  |  |
| N | 21 | 17 | 24 |
| Adjusted Mean (SE) | -7.97 (7.16) | -6.17 (7.66) | -15.36 (6.94) |
| Adjusted CI (a) | -25.16, 9.22 | -24.56, 12.22 | -32.01, 1.30 |
| p-value | 0.557 | 0.766 | 0.078 |
| **Month 6** |  |  |  |
| N | 19 | 14 | 21 |
| Adjusted Mean (SE) | -7.92 (7.74) | -9.82 (8.48) | -8.76 (7.72) |
| Adjusted CI (a) | -26.57, 10.73 | -30.24, 10.59 | -27.36, 9.83 |
| p-value | 0.621 | 0.528 | 0.544 |

CI, confidence interval; JTA-100/2, group of patients receiving an injection of JTA-004 with 100 µg clonidine and 20 mg hyaluronic acid; JTA-200/2, group of patients receiving an injection of JTA-004 with 200 µg clonidine and 20 mg hyaluronic acid; JTA-200/4, group of patients receiving an injection of JTA-004 with 200 µg clonidine and 40 mg hyaluronic acid; N, total number of patients; SE, standard error; WOMAC, Western Ontario McMaster Universities.

(a) calculated using the Dunnett’s test procedure (overall type-I error rate of 0.05).

Differences in adjusted mean change from baseline in WOMAC Total Score were evaluated using an ANCOVA model with treatment group as fixed factor and baseline value of WOMAC Total Score as covariate.

**Supplementary Table 3.** Difference between each JTA group and the reference group in adjusted mean change from baseline in WOMAC Physical Function Subscale Score (interim analysis; Full Analysis Set)

|  | **JTA-100/2** | **JTA-200/2** | **JTA-200/4** |
| --- | --- | --- | --- |
| **Month 3** |  |  |  |
| N | 27 | 23 | 29 |
| Adjusted Mean (SE) | -8.53 (6.39) | -8.04 (6.72) | -13.97 (6.28) |
| Adjusted CI (a) | -23.78, 6.71 | -24.09, 8.00 | -28.97, 1.02 |
| p-value | 0.406 | 0.495 | 0.074 |
| **Month 6** |  |  |  |
| N | 22 | 19 | 24 |
| Adjusted Mean (SE) | -6.02 (7.16) | -12.49 (7.46) | -8.31 (7.08) |
| Adjusted CI (a) | -23.18, 11.13 | -30.36, 5.37 | -25.26, 8.64 |
| p-value | 0.740 | 0.232 | 0.511 |

CI, confidence interval; JTA-100/2, group of patients receiving an injection of JTA-004 with 100 µg clonidine and 20 mg hyaluronic acid; JTA-200/2, group of patients receiving an injection of JTA-004 with 200 µg clonidine and 20 mg hyaluronic acid; JTA-200/4, group of patients receiving an injection of JTA-004 with 200 µg clonidine and 40 mg hyaluronic acid; N, total number of patients; SE, standard error; WOMAC, Western Ontario McMaster Universities.

(a) calculated using the Dunnett’s test procedure (overall type-I error rate of 0.05).

Differences in adjusted mean change from baseline in WOMAC Physical Function Subscale Score were evaluated using an ANCOVA model with treatment group as fixed factor and baseline value of WOMAC Physical Function Subscale Score as covariate.

**Supplementary Table 4.** Difference between each JTA group and the reference group in adjusted mean change from baseline in SF-12 Well-Being Scores (Physical Component Summary Score) (Full Analysis Set)

|  | **JTA-100/2** | **JTA-200/2** | **JTA-200/4** |
| --- | --- | --- | --- |
| **Week 2** | | | |
| **Change from Baseline** |  |  |  |
| N | 39 | 40 | 39 |
| Adjusted Mean (SE), mm | 3.9 (1.3) | 2.6 (1.1) | 2.9 (1.0) |
| **Difference between treatments (JTA-004 minus reference) in Change from Baseline** | | | |
| Adjusted Mean (SE), mm | -0.17 (1.90) | -0.56 (1.55) | 0.26 (1.46) |
| Adjusted CI, mm (a) | -5.03, 4.69 | -4.56, 3.45 | -3.52, 4.03 |
| p-value | 1.000 | 0.996 | 1.000 |
| **Month 3** | | | |
| **Change from Baseline** |  |  |  |
| N | 39 | 34 | 40 |
| Adjusted Mean (SE), mm | 5.4 (1.3) | 4.0 (1.3) | 5.2 (1.2) |
| **Difference between treatments (JTA-004 minus reference) in Change from Baseline** | | | |
| Adjusted Mean (SE), mm | -0.32 (1.84) | -0.73 (1.77) | 0.94 (1.72) |
| Adjusted CI, mm (a) | -5.05, 4.41 | -5.28, 3.83 | -3.46, 5.34 |
| p-value | 1.000 | 0.993 | 0.971 |
| **Month 6** | | | |
| **Change from Baseline** |  |  |  |
| N | 36 | 34 | 40 |
| Adjusted Mean (SE), mm | 6.9 (1.5) | 4.9 (1.5) | 3.7 (1.3) |
| **Difference between treatments (JTA-004 minus reference) in Change from Baseline** | | | |
| Adjusted Mean (SE), mm | 0.30 (2.16) | -0.64 (2.10) | -1.39 (1.87) |
| Adjusted CI, mm (a) | -5.19, 5.79 | -5.96, 4.67 | -6.13, 3.34 |
| p-value | 1.000 | 0.997 | 0.893 |

CI, confidence interval; JTA-100/2, group of patients receiving an injection of JTA-004 with 100 µg clonidine and 20 mg hyaluronic acid; JTA-200/2, group of patients receiving an injection of JTA-004 with 200 µg clonidine and 20 mg hyaluronic acid; JTA-200/4, group of patients receiving an injection of JTA-004 with 200 µg clonidine and 40 mg hyaluronic acid; N, total number of patients; SE, standard error.

(a) calculated using the Dunnett’s test procedure (overall type-I error rate of 0.05).

Changes from baseline in SF-12 Well-Being Scores over time were evaluated using a Mixed-effect Model for Repeated Measurements with absolute change from baseline to the visit in Well-Being Score as response variable, treatment group and visit as factors, baseline Well-Being Score as covariate and treatment group-visit interaction.

**Supplementary Table 5.** Difference between each JTA group and the reference group in adjusted mean change from baseline in SF-12 Well-Being Scores (Mental Component Summary Score) (Full Analysis Set)

|  | **JTA-100/2** | **JTA-200/2** | **JTA-200/4** |
| --- | --- | --- | --- |
| **Week 2** | | | |
| **Change from Baseline** |  |  |  |
| N | 39 | 40 | 39 |
| Adjusted Mean (SE), mm | 0.0 (1.4) | 2.0 (1.4) | 2.1 (1.5) |
| **Difference between treatments (JTA-004 minus reference) in Change from Baseline** | | | |
| Adjusted Mean (SE), mm | -2.98 (1.97) | -0.62 (1.93) | -0.80 (2.05) |
| Adjusted CI (a) | -8.06, 2.10 | -5.60, 4.36 | -6.07, 4.48 |
| p-value | 0.429 | 0.998 | 0.994 |
| **Month 3** | | | |
| **Change from Baseline** |  |  |  |
| N | 39 | 34 | 40 |
| Adjusted Mean (SE), mm | 2.2 (1.5) | -0.0 (1.6) | 1.9 (1.6) |
| **Difference between treatments (JTA-004 minus reference) in Change from Baseline** | | | |
| Adjusted Mean (SE), mm | 0.48 (2.16) | -1.29 (2.25) | 0.35 (2.28) |
| Adjusted CI (a) | -5.06, 6.03 | -7.07, 4.48 | -5.48, 6.18 |
| p-value | 1.000 | 0.968 | 1.000 |
| **Month 6** | | | |
| **Change from Baseline** |  |  |  |
| N | 36 | 34 | 40 |
| Adjusted Mean (SE), mm | 1.7 (1.8) | 0.1 (1.8) | 0.2 (1.6) |
| **Difference between treatments (JTA-004 minus reference) in Change from Baseline** | | | |
| Adjusted Mean (SE), mm | 1.23 (2.50) | 0.03 (2.43) | -0.11 (2.31) |
| Adjusted CI (a) | -5.12, 7.58 | -6.14, 6.21 | -6.00, 5.79 |
| p-value | 0.979 | 1.000 | 1.000 |

CI, confidence interval; JTA-100/2, group of patients receiving an injection of JTA-004 with 100 µg clonidine and 20 mg hyaluronic acid; JTA-200/2, group of patients receiving an injection of JTA-004 with 200 µg clonidine and 20 mg hyaluronic acid; JTA-200/4, group of patients receiving an injection of JTA-004 with 200 µg clonidine and 40 mg hyaluronic acid; N, total number of patients; SE, standard error.

(a) calculated using the Dunnett’s test procedure (overall type-I error rate of 0.05).

Changes from baseline in SF-12 Well-Being Scores over time were evaluated using a Mixed-effect Model for Repeated Measurements with absolute change from baseline to the visit in Well-Being Score as response variable, treatment group and visit as factors, baseline Well-Being Score as covariate and treatment group x visit interaction.

**Supplementary Table 6.** Consumption of analgesics - Values by visit and absolute change from baseline (Full Analysis Set)

|  | **JTA-100/2** | **JTA-200/2** | **JTA-200/4** | **reference** |
| --- | --- | --- | --- | --- |
| **Week 2** | | | | |
| N | 40 | 41 | 41 | 41 |
| Mean ± SD | 0.225 ± 0.530 | 0.171 ± 0.381 | 0.146 ± 0.358 | 0.220 ± 0.475 |
| **Change from Baseline** | | | | |
| No change | 24 (60.0%) | 21 (51.2%) | 29 (70.7%) | 25 (61.0%) |
| Decrease | 12 (30.0%) | 19 (46.3%) | 12 (29.3%) | 12 (29.3%) |
| Increase | 4 (10.0%) | 1 (2.4%) | 0 (0.0%) | 4 (9.8%) |
| **Month 3** | | | | |
| N | 39 | 36 | 41 | 40 |
| Mean ± SD | 0.308 ± 0.694 | 0.333 ± 0.717 | 0.171 ± 0.442 | 0.350 ± 0.580 |
| **Change from Baseline** | | | | |
| No change | 27 (69.2%) | 23 (63.9%) | 34 (82.9%) | 27 (67.5%) |
| Decrease | 9 (23.1%) | 6 (16.7%) | 5 (12.2%) | 10 (25.0%) |
| Increase | 3 (7.7%) | 7 (19.4%) | 2 (4.9%) | 3 (7.5%) |
| **Month 6** | | | | |
| N | 36 | 35 | 40 | 39 |
| Mean ± SD | 0.278 ± 0.513 | 0.457 ± 1.039 | 0.325 ± 0.572 | 0.385 ± 0.782 |
| **Change from Baseline** | | | | |
| No change | 25 (69.4%) | 23 (65.7%) | 28 (70.0%) | 30 (76.9%) |
| Decrease | 4 (11.1%) | 8 (22.9%) | 5 (12.5%) | 2 (5.1%) |
| Increase | 7 (19.4%) | 4 (11.4%) | 7 (17.5%) | 7 (17.9%) |

JTA-100/2, group of patients receiving an injection of JTA-004 with 100 µg clonidine and 20 mg hyaluronic acid; JTA-200/2, group of patients receiving an injection of JTA-004 with 200 µg clonidine and 20 mg hyaluronic acid; JTA-200/4, group of patients receiving an injection of JTA-004 with 200 µg clonidine and 40 mg hyaluronic acid; N, total number of patients; reference, group of patients receiving an injection of the reference treatment (hylan G-F 20); SD, standard deviation.

**Supplementary Table 7.** Consumption of NSAIDs - Values by visit and absolute change from baseline (Full Analysis Set)

|  | **JTA-100/2** | **JTA-200/2** | **JTA-200/4** | **reference** |
| --- | --- | --- | --- | --- |
| **Week 2** | | | | |
| N | 40 | 41 | 41 | 41 |
| Mean ± SD | 0.200 ± 0.464 | 0.146 ± 0.358 | 0.220 ± 0.525 | 0.098 ± 0.374 |
| **Change from Baseline** | | | | |
| No change | 27 (67.5%) | 26 (63.4%) | 31 (75.6%) | 29 (70.7%) |
| Decrease | 12 (30.0%) | 13 (31.7%) | 9 (22.0%) | 11 (26.8%) |
| Increase | 1 (2.5%) | 2 (4.9%) | 1 (2.4%) | 1 (2.4%) |
| **Month 3** | | | | |
| N | 38 | 36 | 41 | 40 |
| Mean ± SD | 0.154 ± 0.432 | 0.361 ± 0.762 | 0.073 ± 0.264 | 0.375 ± 0.774 |
| **Change from Baseline** | | | | |
| No change | 30 (78.9%) | 23 (63.9%) | 34 (82.9%) | 26 (65.0%) |
| Decrease | 6 (15.8%) | 6 (16.7%) | 7 (17.1%) | 6 (15.0%) |
| Increase | 2 (5.3%) | 7 (19.4%) | 0 (0.0%) | 8 (20.0%) |
| **Month 6** | | | | |
| N | 36 | 35 | 40 | 39 |
| Mean ± SD | 0.167 ± 0.697 | 0.314 ± 1.051 | 0.300 ± 0.608 | 0.231 ± 0.536 |
| **Change from Baseline** | | | | |
| No change | 29 (80.6%) | 29 (82.9%) | 30 (75.0%) | 30 (76.9%) |
| Decrease | 5 (13.9%) | 4 (11.4%) | 2 (5.0%) | 7 (17.9%) |
| Increase | 2 (5.6%) | 2 (5.7%) | 8 (20.0%) | 2 (5.1%) |

JTA-100/2, group of patients receiving an injection of JTA-004 with 100 µg clonidine and 20 mg hyaluronic acid; JTA-200/2, group of patients receiving an injection of JTA-004 with 200 µg clonidine and 20 mg hyaluronic acid; JTA-200/4, group of patients receiving an injection of JTA-004 with 200 µg clonidine and 40 mg hyaluronic acid; N, total number of patients; NSAID, non-steroidal anti-inflammatory drug; reference, group of patients receiving an injection of the reference treatment (hylan G-F 20); SD, standard deviation.

**Supplementary Table 8.** Difference between the pooled JTA group and the reference group in adjusted mean change from baseline in WOMAC Total Score over time (Full Analysis Set)

|  | **Pooled JTA**  **(N=123)** | **Reference**  **(N=41)** | **Difference between treatments (JTA minus reference)** |
| --- | --- | --- | --- |
| **Month 3** | | | |
| **Change from Baseline** |  |  |  |
| N | 113 | 40 |  |
| Adjusted Mean (SE) | -26.3 (2.2) | -19.7 (3.7) | -6.62 (4.30) |
| Adjusted CI (a) |  |  | -15.11, 1.87 |
| p-value |  |  | 0.126 |
| **Month 6** | | | |
| **Change from Baseline** |  |  |  |
| N | 111 | 39 |  |
| Adjusted Mean (SE) | -26.2 (2.4) | -17.3 (4.1) | -8.88 (4.76) |
| Adjusted CI (a) |  |  | -28.28, 0.52 |
| p-value |  |  | 0.064 |

CI, confidence interval; N, total number of patients; pooled JTA, group of patients receiving an injection of any formulation of JTA-004; reference, group of patients receiving an injection of the reference treatment (hylan G-F 20); SE, standard error; WOMAC, Western Ontario McMaster Universities.

(a) Calculated using Dunnett-corrected t-value.

Differences in adjusted mean change from baseline in WOMAC Total Score were evaluated using an ANCOVA model with treatment group as fixed factor and baseline value of WOMAC Total Score as covariate.

**Supplementary Table 9.** Adverse events related to study procedures (Safety Set)

|  | **JTA-100/2 (N=41)** | | **JTA-200/2 (N=41)** | | **JTA-200/4 (N=41)** | | **reference (N=41)** | |
| --- | --- | --- | --- | --- | --- | --- | --- | --- |
|  | **m** | **n (%)** | **m** | **n (%)** | **m** | **n (%)** | **m** | **n (%)** |
| At least one AE related to study procedure | 4 | 3 (7.3) | 10 | 6 (14.6) | 12 | 9 (22.0) | 10 | 8 (19.5) |
| General Disorders and Administration Site Conditions | 0 | 0 (0.0) | 2 | 2 (4.9) | 3 | 3 (7.3) | 2 | 2 (4.9) |
| Injection Site Pain | 0 | 0 (0.0) | 0 | 0 (0.0) | 1 | 1 (2.4) | 2 | 2 (4.9) |
| Application Site Edema | 0 | 0 (0.0) | 1 | 1 (2.4) | 0 | 0 (0.0) | 0 | 0 (0.0) |
| Condition Aggravated | 0 | 0 (0.0) | 1 | 1 (2.4) | 0 | 0 (0.0) | 0 | 0 (0.0) |
| Fatigue | 0 | 0 (0.0) | 0 | 0 (0.0) | 1 | 1 (2.4) | 0 | 0 (0.0) |
| Thirst | 0 | 0 (0.0) | 0 | 0 (0.0) | 1 | 1 (2.4) | 0 | 0 (0.0) |
| Musculoskeletal and Connective Tissue Disorders | 1 | 1 (2.4) | 1 | 1 (2.4) | 2 | 2 (4.9) | 4 | 3 (7.3) |
| Arthralgia | 0 | 0 (0.0) | 1 | 1 (2.4) | 0 | 0 (0.0) | 4 | 3 (7.3) |
| Joint Stiffness | 1 | 1 (2.4) | 0 | 0 (0.0) | 0 | 0 (0.0) | 0 | 0 (0.0) |
| Pain in Extremity | 0 | 0 (0.0) | 0 | 0 (0.0) | 1 | 1 (2.4) | 0 | 0 (0.0) |
| Tendonitis | 0 | 0 (0.0) | 0 | 0 (0.0) | 1 | 1 (2.4) | 0 | 0 (0.0) |
| Injury, Poisoning and Procedural Complications | 1 | 1 (2.4) | 1 | 1 (2.4) | 3 | 3 (7.3) | 1 | 1 (2.4) |
| Procedural Hypotension | 0 | 0 (0.0) | 0 | 0 (0.0) | 3 | 3 (7.3) | 0 | 0 (0.0) |
| Procedural Pain | 1 | 1 (2.4) | 0 | 0 (0.0) | 0 | 0 (0.0) | 1 | 1 (2.4) |
| Delayed Recovery from Anesthesia | 0 | 0 (0.0) | 1 | 1 (2.4) | 0 | 0 (0.0) | 0 | 0 (0.0) |
| Skin and Subcutaneous Tissue Disorders | 1 | 1 (2.4) | 0 | 0 (0.0) | 2 | 2 (4.9) | 0 | 0 (0.0) |
| Hyperkeratosis | 1 | 1 (2.4) | 0 | 0 (0.0) | 0 | 0 (0.0) | 0 | 0 (0.0) |
| Skin Irritation | 0 | 0 (0.0) | 0 | 0 (0.0) | 1 | 1 (2.4) | 0 | 0 (0.0) |
| Skin Lesion | 0 | 0 (0.0) | 0 | 0 (0.0) | 1 | 1 (2.4) | 0 | 0 (0.0) |
| Gastrointestinal Disorders | 0 | 0 (0.0) | 1 | 1 (2.4) | 0 | 0 (0.0) | 1 | 1 (2.4) |
| Melaena | 0 | 0 (0.0) | 0 | 0 (0.0) | 0 | 0 (0.0) | 1 | 1 (2.4) |
| Nausea | 0 | 0 (0.0) | 1 | 1 (2.4) | 0 | 0 (0.0) | 0 | 0 (0.0) |
| Investigations | 0 | 0 (0.0) | 1 | 1 (2.4) | 0 | 0 (0.0) | 1 | 1 (2.4) |
| Blood Creatine Phosphokinase Increased | 0 | 0 (0.0) | 0 | 0 (0.0) | 0 | 0 (0.0) | 1 | 1 (2.4) |
| Blood Pressure Decreased | 0 | 0 (0.0) | 1 | 1 (2.4) | 0 | 0 (0.0) | 0 | 0 (0.0) |
| Nervous System Disorders | 0 | 0 (0.0) | 1 | 1 (2.4) | 1 | 1 (2.4) | 0 | 0 (0.0) |
| Headache | 0 | 0 (0.0) | 0 | 0 (0.0) | 1 | 1 (2.4) | 0 | 0 (0.0) |
| Presyncope | 0 | 0 (0.0) | 1 | 1 (2.4) | 0 | 0 (0.0) | 0 | 0 (0.0) |
| Surgical and Medical Procedures | 1 | 1 (2.4) | 0 | 0 (0.0) | 0 | 0 (0.0) | 1 | 1 (2.4) |
| Joint Injection | 1 | 1 (2.4) | 0 | 0 (0.0) | 0 | 0 (0.0) | 1 | 1 (2.4) |
| Infections and Infestations | 0 | 0 (0.0) | 3 | 1 (2.4) | 0 | 0 (0.0) | 0 | 0 (0.0) |
| Osteomyelitis Acute | 0 | 0 (0.0) | 1 | 1 (2.4) | 0 | 0 (0.0) | 0 | 0 (0.0) |
| Osteomyelitis Chronic | 0 | 0 (0.0) | 2 | 1 (2.4) | 0 | 0 (0.0) | 0 | 0 (0.0) |
| Vascular Disorders | 0 | 0 (0.0) | 0 | 0 (0.0) | 1 | 1 (2.4) | 0 | 0 (0.0) |
| Hypotension | 0 | 0 (0.0) | 0 | 0 (0.0) | 1 | 1 (2.4) | 0 | 0 (0.0) |

AE, adverse event; m, number of serious adverse events; n, number of patients with at least one serious AE; JTA-100/2, group of patients receiving an injection of JTA-004 with 100 µg clonidine and 20 mg hyaluronic acid; JTA-200/2, group of patients receiving an injection of JTA-004 with 200 µg clonidine and 20 mg hyaluronic acid; JTA-200/4, group of patients receiving an injection of JTA-004 with 200 µg clonidine and 40 mg hyaluronic acid; N, total number of patients; reference, group of patients receiving an injection of the reference treatment (hylan G-F 20); %, (n row / N group) x 100.
