# Supplementary material for: Safety and efficacy of a single intra-articular injection of a novel enhanced protein solution (JTA-004) compared to hylan G-F 20 in symptomatic knee osteoarthritis: a randomized, double-blind, controlled phase II/III study
Source: BMC Musculoskelet Disord. 2021 Oct 19;22:888. doi: 10.1186/s12891-021-04750-3 (PMC8527807; doi:10.1186/s12891-021-04750-3)
Supplement: Supplementary file 3 — Additional file 3: Supplementary Fig. 1. Change from baseline in WOMAC (A) Pain Subscale and (B) Physical Function Subscale Score over time in the JTA-100/2 group and in the reference group (Full Analysis Set). Supplementary Fig. 2. Change from baseline in WOMAC (A) Pain Subscale and (B) Physical Function Subscale Score over time in the JTA-200/4 group and in the reference group (Full Analysis Set). Supplementary Fig. 3. Difference between the pooled JTA-004 group and the reference group in adjusted mean change from baseline in WOMAC Physical Function Subscale Score at Month 3 and Month 6 (Full Analysis Set). [file 12891_2021_4750_MOESM3_ESM.docx]

**Additional file 3. Supplementary figures**

**Supplementary Figure 1.** Change from baseline in WOMAC (a) Pain Subscale and (b) Physical Function Subscale Score over time in the JTA-100/2 group and in the reference group (Full Analysis Set)


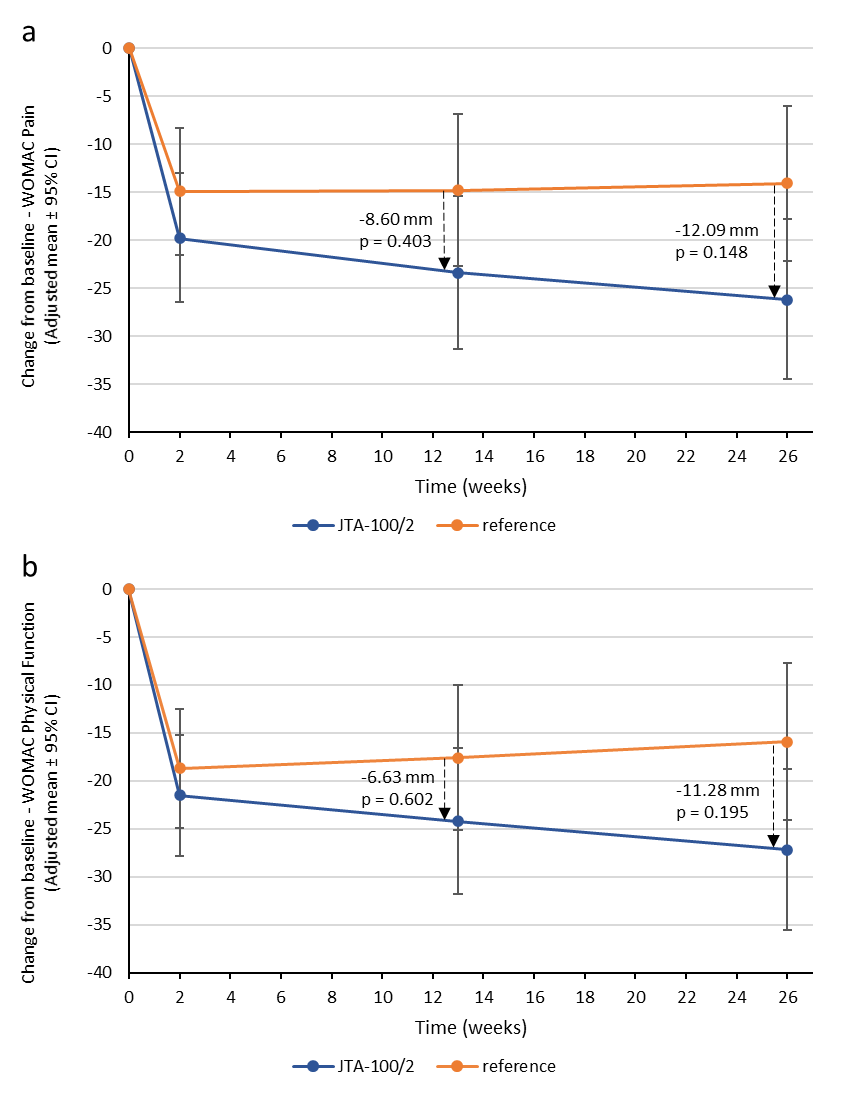


Footnote: CI, confidence interval (calculated using Dunnett-corrected t-value); JTA-100/2, group of patients receiving an injection of JTA-004 with 100 µg clonidine and 20 mg hyaluronic acid; reference, group of patients receiving an injection of the reference treatment (hylan G-F 20); WOMAC, Western Ontario McMaster Universities. Changes from baseline in WOMAC Subscale Scores over time were evaluated using a Mixed-effect Model for Repeated Measurements with absolute change from baseline to the visit in WOMAC Subscale Score as response variable, treatment group and visit as factors, baseline WOMAC Subscale Score as covariate and treatment group-visit interaction.

**Supplementary Figure 2.** Change from baseline in WOMAC (a) Pain Subscale and (b) Physical Function Subscale Score over time in the JTA-200/4 group and in the reference group (Full Analysis Set)


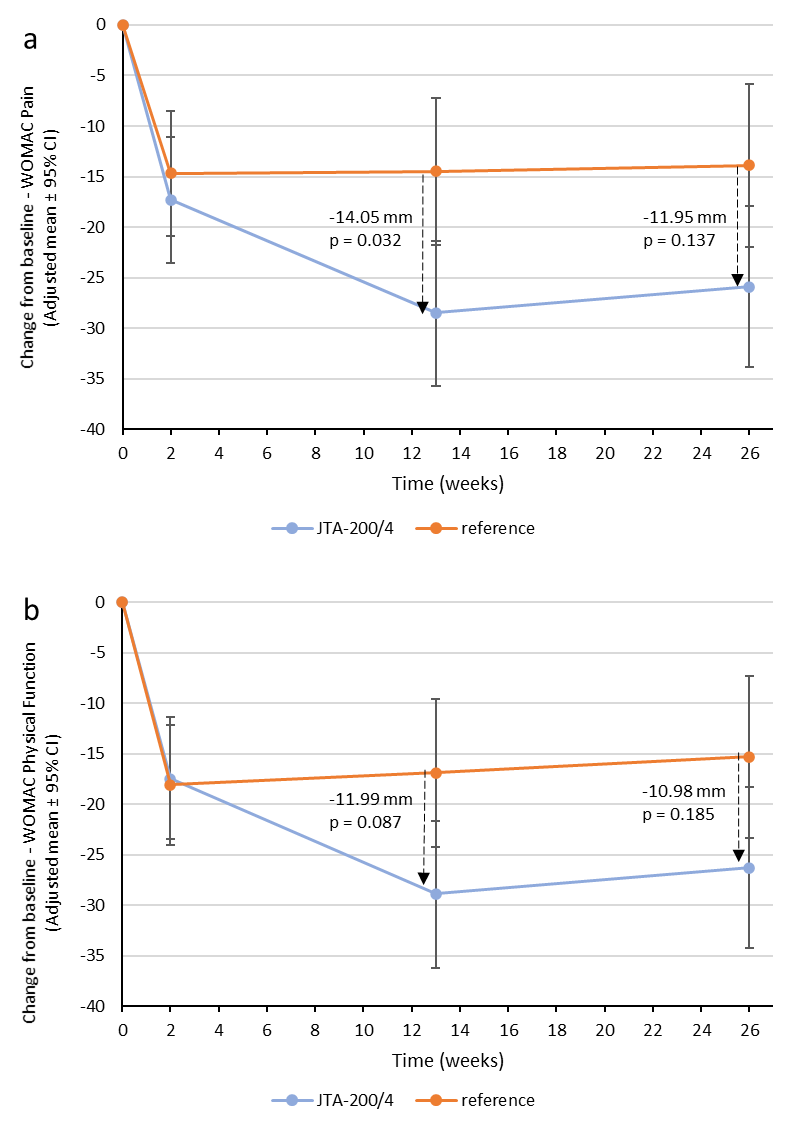


Footnote: CI, confidence interval (calculated using Dunnett-corrected t-value); JTA-200/4, group of patients receiving an injection of JTA-004 with 200 µg clonidine and 40 mg hyaluronic acid; reference, group of patients receiving an injection of the reference treatment (hylan G-F 20); WOMAC, Western Ontario McMaster Universities. Changes from baseline in WOMAC Subscale Scores over time were evaluated using a Mixed-effect Model for Repeated Measurements with absolute change from baseline to the visit in WOMAC Subscale Score as response variable, treatment group and visit as factors, baseline WOMAC Subscale Score as covariate and treatment group x visit interaction.

**Supplementary Figure 3.** Difference between the pooled JTA-004 group and the reference group in adjusted mean change from baseline in WOMAC Physical Function Subscale Score at Month 3 and Month 6 (Full Analysis Set)


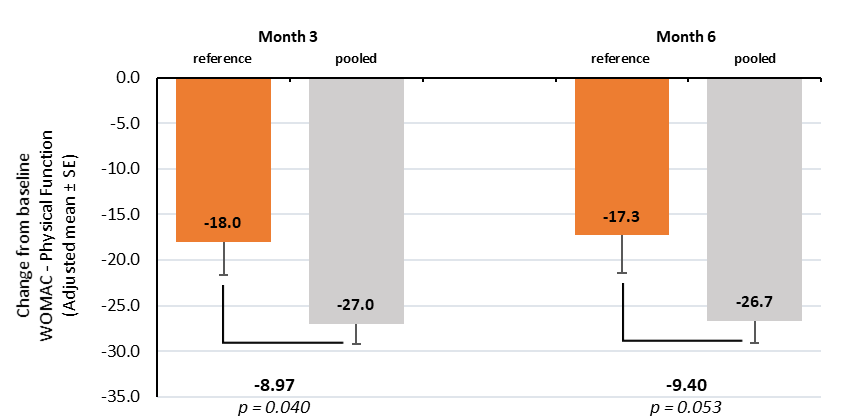


Footnote: pooled, group of patients receiving an injection of any formulation of JTA-004; reference, group of patients receiving an injection of the reference treatment (hylan G-F 20); SE, standard error; WOMAC, Western Ontario McMaster Universities. Differences in adjusted mean change from baseline in WOMAC Physical Function Subscale Score were evaluated using an ANCOVA model with treatment group as fixed factor and baseline value of WOMAC Physical Function Subscale Score as covariate.
